# Gold-catalyzed direct alkynylation of tryptophan in peptides using TIPS-EBX

**DOI:** 10.3762/bjoc.12.74

**Published:** 2016-04-19

**Authors:** Gergely L Tolnai, Jonathan P Brand, Jerome Waser

**Affiliations:** 1 Laboratory of Catalysis and Organic Synthesis, Ecole Polytechnique Fédérale de Lausanne, EPFL SB ISIC LCSO, BCH 4306, 1015 Lausanne, Switzerland; 2 Department of Organic Chemistry, Arrhenius Laboratory, Stockholm University, SE-106 91 Stockholm, Sweden; 3 Givaudan, Chemin de la parfumerie 5, 1214 Vernier, Switzerland

**Keywords:** alkynes, C–H functionalization, gold catalysis, hypervalent iodine, peptides

## Abstract

The selective functionalization of peptides containing only natural amino acids is important for the modification of biomolecules. In particular, the installation of an alkyne as a useful handle for bioconjugation is highly attractive, but the use of a carbon linker is usually required. Herein, we report the gold-catalyzed direct alkynylation of tryptophan in peptides using the hypervalent iodine reagent TIPS-EBX (1-[(triisopropylsilyl)ethynyl]-1,2-benziodoxol-3(1*H*)-one). The reaction proceeded in 50–78% yield under mild conditions and could be applied to peptides containing other nucleophilic and aromatic amino acids, such as serine, phenylalanine or tyrosine.

## Introduction

**Figure 1:**
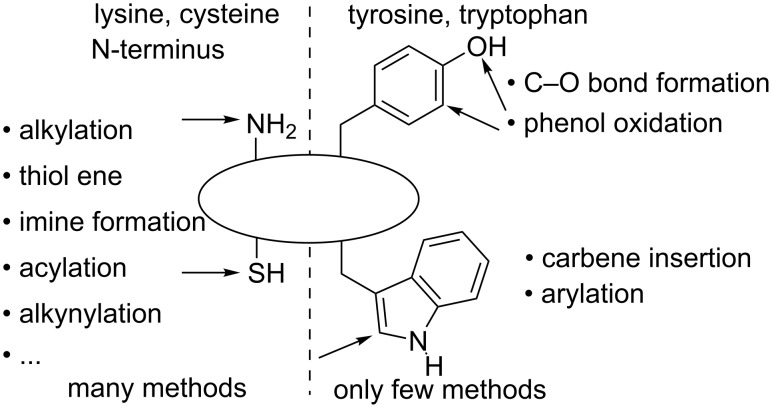
Functionalization of natural peptides and proteins: state of the art.

Alkynes have always been important building blocks in synthetic organic chemistry. Recently, they have attracted also strong interest for applications in materials science and chemical biology [1]. One of the most important transformations of alkynes is the copper-catalyzed [3 + 2] cycloaddition with azides, which can be performed under mild conditions in the presence of multiple functional groups, and has therefore found broad applications for the modification of biomolecules and polymers [2–5]. But before the unique reactivity of the triple bond can be unravelled, it is necessary to introduce it onto the desired molecules. In this context, the modification of natural peptides and proteins is highly attractive, and it has been the target of intensive research in the last decades (Figure 1) [6–11]. The functionalization of highly reactive cysteine, lysine and the N-terminus has been particularly successful [12–17], whereas the more challenging modification of the electron-rich aromatic residues of tyrosine [18–20] and tryptophan [21–31] has been the focus of recent interest. As tryptophan is a rare amino acid, its functionalization is especially interesting. It has been achieved in the past for example by Francis and co-workers and Ball and co-workers using rhodium-catalyzed carbene-insertion reactions [21–23] or via direct C–H arylation [24–29]. If the installation of alkynes on peptides or proteins is desired, an indirect method using a linker is used, for example an alkylation reaction of cysteine. The direct introduction of an alkyne onto the biomolecule would be interesting to profit from modified electronic and spectroscopic properties. However, the direct alkynylation of peptides or proteins is usually based on the use of the Sonogashira reaction, which requires modified non-natural amino acids [32–33].

**Scheme 1:**
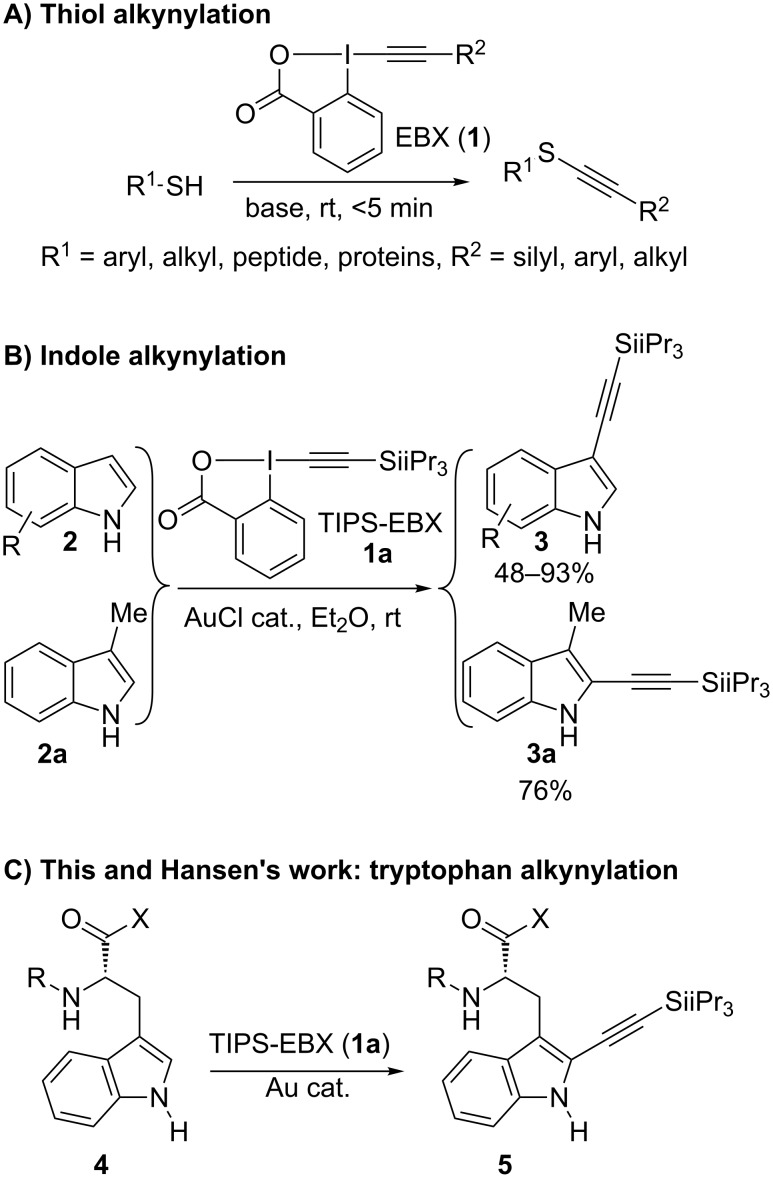
Alkynylation with EBX reagents.

In 2013, our group reported the alkynylation of thiols using the hypervalent iodine reagent TIPS-EBX (**1a**, 1-[(triisopropylsilyl)ethynyl]-1,2-benziodoxol-3(1*H*)-one) (Scheme 1A) [34]. The reaction was almost instantaneous. It was highly chemoselective for thiols in the presence of other nucleophilic functional groups. The alkynylation could be therefore applied to cysteine-containing peptides. The scope of the reaction could be later extended to a broad range of aliphatic and aromatic alkynes [35]. In 2015, the efficiency of the reaction for the functionalization of proteins both in cell lysates and in the living cell was finally demonstrated [36].

Even if the alkynylation of cysteines is an important method, thiols are often part of disulfide bonds in folded proteins, and therefore difficult to access. Reduction and unfolding, or protein engineering to install more accessible cysteines, are usually required. For these reasons, it is important to develop selective alkynylation methods in order to functionalize other amino acids. The direct C–H functionalization of aromatic compounds is an attractive method for the modification of biomolecules without the need for non-natural amino acids. However, the multiple functional groups present in biomolecules make such a process highly challenging. Based on our previous work on the alkynylation of indoles using TIPS-EBX (**1a**) and a gold catalysis [37–38], we wondered if this transformation could be extended to tryptophan-containing peptides. Even if the reaction gave C3-alkynylation for C3-unsubstituted indoles, we demonstrated that C2-alkynylation could be achieved on skatole (**2a**, Scheme 1B) [37]. Very recently, Hansen et al. indeed reported a modified protocol using a gold catalyst and TIPS-EBX (**1a**) for the alkynylation of tryptophan-containing peptides and even proteins (Scheme 1C) [39]. This recent disclosure motivated us to report our own work on this transformation, resulting in an efficient direct alkynylation of tryptophan-containing peptides.

## Results and Discussion

We started our investigation by attempting the alkynylation of valine-tryptophan dipeptide **4a** as model substrate (Table 1). An often used carboxybenzyl (Cbz, Z) protecting group was chosen. Examining this substrate will tell if C2-alkynylation is possible in the presence of an ester, a carbamate and an amide protecting group. A promising result was obtained with 5 mol % gold chloride as catalyst at room temperature in acetonitrile (Table 1, entry 1). Although the reaction did not go to completion even after two days, the desired C2 alkynylation product **5a** was obtained in 44% yield. The yield could be increased to 72% when the reaction was performed at 40 °C (Table 1, entry 2). No further improvement was observed at higher temperature (Table 1, entry 3). The product **5a** could also be obtained in a broad range of other solvents, as long as the solubility of the substrate **4a** and TIPS-EBX (**1a**) was sufficient (Table 1, entries 4–8). The best yield was obtained in acetonitrile (Table 1, entry 2). Although the presence of water slowed down the reaction, the desired product could still be obtained in 41% yield (Table 1, entry 9). Monitoring the reaction over time showed that 34% of product **5a** was already formed after 20 min (Table 1, entry 10), but the reaction then slowed down significantly, with 67% yield after 10 h and 78% after 24 h (Table 1, entries 11 and 12). At this point, a conversion higher than 90% was achieved, with no significant improvement after a longer reaction time.

**Table 1:** Optimization of the alkynylation of dipeptide **4a**.

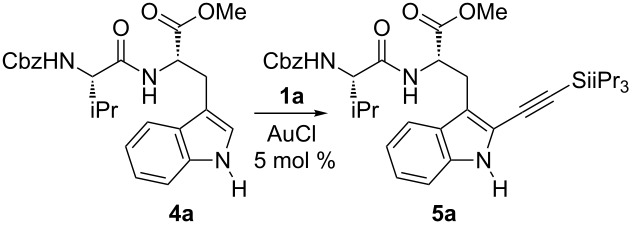				

entry	solvent	time (h)	*T* (°C)	yield^a^

1	CH_3_CN	48	23	44%
2	CH_3_CN	48	40	72%
3	CH_3_CN	48	60	67%
4	iPrOH	48	40	60%
5	MeOH	48	40	50%
6	acetone	48	40	39%
7	CH_2_Cl_2_	48	40	63%
8	DMSO	48	40	38%
9	CH_3_CN 5% H_2_O	48	40	41%
10	CH_3_CN	0.3	40	34%
11	CH_3_CN	10	40	67%
12	CH_3_CN	24	40	78%

^a^Reaction conditions: 0.20 mmol **4a**, 0.24 mmol TIPS-EBX (**1a**), 0.010 mmol AuCl in 2 mL solvent were stirred at the indicated temperature and time. Isolated yields after column chromatography are given.

**Scheme 2:**
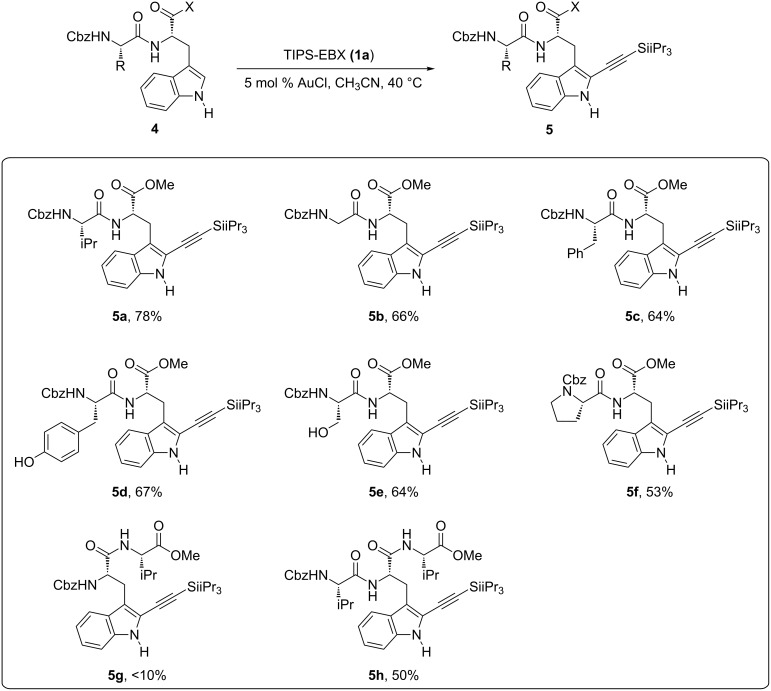
Alkynylation of tryptophan-containing peptides.

With the optimized conditions in hand, we investigated the scope of the reaction with different amino acids in the dipeptide (Scheme 2). With glycine as second amino acid, the desired product **5b** could be obtained in 66% yield. The reaction was selective for tryptophan in the presence of other aromatic amino acids, such as phenylalanine or tyrosine (products **5c** and **5d**). Serine and proline containing dipeptides **5e** and **5f** could also be obtained in 64% and 53% yield, respectively. The reaction was therefore general for dipeptides bearing tryptophan at the C-terminus. On the other hand, only traces of alkynylated dipeptide **5g** with a tryptophan at the N-terminus could be obtained under these reaction conditions. A first example of valine–tryptophan–valine tripeptide was also examined, and product **5h** was isolated in 50% yield, demonstrating that alkynylation of tryptophan inside a peptide chain was possible. Unfortunately, only limited conversion was observed with N- or C-terminus unprotected peptides. Nevertheless, Hansen and co-workers recently demonstrated that N- and C-termini unprotected peptides, as well as more complex peptides and even proteins, could be alkynylated using modified reaction conditions (10 mol % AuCl(SMe_2_), three equivalents TIPS-EBX (**1a**) and 2 mol % trifluoroacetic acid as co-catalyst) [39]. They also demonstrated that the obtained silylalkyne products can be easily deprotected with fluoride sources to allow bioconjugation via cycloaddition with azides.

## Conclusion

In conclusion, our work combined with the results of Hansen and co-workers has demonstrated that the gold-catalyzed alkynylation of indoles could be extended to tryptophan in peptides. When considering the scarcity of methods allowing the modification of tryptophan under mild conditions without requiring the installation of non-natural amino acids, the transformation will be highly useful for bioconjugation. A current limitation of the developed alkynylation reaction is the requirement for organic solvents. Investigations are currently ongoing in our laboratory for the development of water-compatible reagents and catalysts.

## Experimental

### General procedure for the gold-catalyzed alkynylation

The starting peptide **4** (0.20 mmol, 1 equiv) and TIPS-EBX (**1a**, 0.240 mmol, 103 mg, 1.2 equiv) were added into a 5 mL test tube equipped with a stirring bar. Acetonitrile (2 mL) was added, then the reaction mixture was stirred at 40 °C for 2 min. Gold(I) chloride (2.3 mg, 10 µmol, 0.05 equiv) was added in one portion. The reaction tube was sealed and stirring was continued for 24 h at 40 °C. Afterwards, the mixture was diluted with EtOAc (50 mL), and the organic layer was washed with a mixture of water (2.5 mL) and conc. NaHCO_3_ solution (2.5 mL), and then with brine (20 mL), and dried over MgSO_4_. The solvent was evaporated under reduced pressure and the resulting yellow oil was purified by column chromatography (SiO_2_, hexane/EtOAc 3:1 to 2:3). The product was dried under reduced pressure, and washed into a vial with Et_2_O. The solvent was evaporated under vacuum and dried under high vacuum (ca. 10^−2^ mbar) for several hours.

## Supporting Information

File 1 Experimental procedure and characterization data for all compounds. NMR spectra of new compounds.
