# Supplementary material for: Gold-catalyzed direct alkynylation of tryptophan in peptides using TIPS-EBX
Source: Beilstein J Org Chem. 2016 Apr 19;12:745–9. doi: 10.3762/bjoc.12.74 (PMC4902029; doi:10.3762/bjoc.12.74)
Supplement: File 1 — Experimental procedure and characterization data for all compounds. NMR spectra of new compounds. [file Beilstein_J_Org_Chem-12-745-s001.pdf]

Supporting Information

for

Gold-catalyzed direct alkynylation of tryptophan in peptides using TIPS-EBX

Gergely L. Tolnai[1,2], Jonathan P. Brand[1,3] and Jerome Waser[1*]

Address: [1]Laboratory of Catalysis and Organic Synthesis, Ecole Polytechnique Fédérale de Lausanne, EPFL SB ISIC LCSO, BCH 4306, 1015 Lausanne, Switzerland, [2]Present address: Department of Organic Chemistry, Arrhenius Laboratory, Stockholm University, SE-106 91 Stockholm, Sweden and [3]Present address: Givaudan, Chemin de la parfumerie 5, 1214 Vernier, Switzerland

Email: Jérôme Waser - jerome.waser@epfl.ch

* Corresponding author

Experimental procedure and characterization data for all compounds.

NMR spectra of new compounds

Table of Contents

1. General methods S2

2. Preparation of TIPS-EBX (**1a**) S3

3. Preparation of peptides S6

4. Alkynylation of peptides S16

5. Spectra for new compounds S23

1. General methods

All reactions were carried out in oven-dried glassware under an atmosphere of nitrogen, unless stated otherwise. For quantitative flash chromatography technical grade solvents were used. For flash chromatography for analysis, HPLC grade solvents from Sigma-Aldrich were used. THF, Et_2O, CH_3CN, toluene, hexane and CH_2Cl_2 were dried by passage over activated alumina under nitrogen atmosphere (H_2O content < 10 ppm, Karl-Fischer titration). All chemicals were purchased from Acros, Aldrich, Fluka, VWR, Aplichem or Merck and used as such unless stated otherwise. Chromatographic purification was performed as flash chromatography using Macherey-Nagel silica 40–63, 60 Å, using the solvents indicated as eluent with 0.1–0.5 bar pressure. TLC was performed on Merck silica gel 60 F_{254} TLC glass plates or aluminium plates and visualized with UV light, permanganate stain, CAN stain or anisaldehyde stain. Melting points were measured on a calibrated Büchi B-540 melting point apparatus using open glass capillaries. ^{1}H NMR spectra were recorded on a Bruker DPX-400 400 MHz spectrometer in chloroform-d, DMSO-d_6 or CD_3OD, all signals are reported in ppm with the internal chloroform signal at 7.26 ppm or the internal DMSO signal at 2.50 ppm as standard. The data is being reported as (s = singlet, d = doublet, t = triplet, q = quadruplet, qi = quintet, m = multiplet or unresolved, br = broad signal, app = apparent, coupling constant(s) in Hz, integration, interpretation).^{13}C NMR spectra were recorded with ^{1}H-decoupling on a Brucker DPX-400 100 MHz spectrometer in chloroform-d, DMSO-d_6, all signals are reported in ppm with the internal chloroform signal at 77.0 ppm, the internal DMSO signal at 39.5 ppm. Infrared spectra were recorded on a JASCO FT-IR B4100 spectrophotometer with an ATR PRO410-S and a ZnSe prisma and are reported as cm^{-1} (w = weak, m = medium, s = strong, br = broad). High resolution mass spectrometric measurements were performed by the mass spectrometry service of ISIC at the EPFL on a MICROMASS (ESI) Q-TOF Ultima API.

2. Preparation of TIPS-EBX (1a)

The synthesis of TIPS-EBX (**1a**) has been already described before. The procedures are taken here from the indicated publications to facilitate reproduction of the results by having all the data in the same file. This reagent is also commercially available.

1-Hydroxy-1,2-benziodoxol-3-(1H)-one (7)

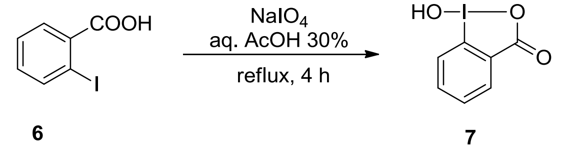

Following a reported procedure,[1] $NaIO_4$ (7.24 g, 33.8 mmol, 1.05 equiv) and 2-iodobenzoic acid (**6**) (8.00 g, 32.2 mmol, 1.00 equiv) were suspended in 30% (v/v) aq. AcOH (48 mL). The mixture was vigorously stirred and refluxed for 4 h. The reaction mixture was then diluted with cold water (180 mL) and allowed to cool to room temperature, protecting it from light. After 1 h, the crude product was collected by filtration, washed on the filter with ice cold water (3 x 20 mL) and acetone (3 x 20 mL), and air-dried in the dark to give the pure product **7** (8.3 g, 31 mmol, 98%) as a white solid. ^{1}H NMR (400 MHz, $(CD_3)_2SO$): δ 8.02 (dd, *J* = 7.7, 1.4 Hz, 1H, Ar*H*), 7.97 (m, 1H, Ar*H*), 7.85 (dd, *J* = 8.2, 0.7 Hz, 1H, Ar*H*), 7.71 (td, *J* = 7.6, 1.2 Hz, 1H, ArH); ^{13}C NMR (100 MHz, $(CD_3)_2SO$): δ 167.7, 134.5, 131.5, 131.1, 130.4, 126.3, 120.4; IR ν 3083 (w), 3060 (w), 2867 (w), 2402 (w), 1601 (m), 1585 (m), 1564 (m), 1440 (m), 1338 (s), 1302 (m), 1148 (m), 1018 (w), 834 (m), 798 (w), 740 (s), 694 (s), 674 (m), 649 (m). The values of the NMR spectra are in accordance with reported literature data.[1]

Triisopropylsilyl(trimethylsilyl)acetylene (9)

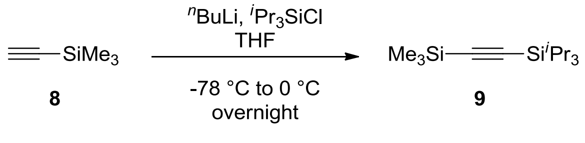

Following a reported procedure,[2] *n*-BuLi (2.5 M in hexanes, 12.0 mL, 29.9 mmol, 0.98 equiv) was added dropwise to a stirred solution of ethynyltrimethylsilane (**8**, 3.0 g, 30 mmol, 1.0 equiv) in THF (48 mL) at −78 °C. The mixture was then warmed to 0 °C and stirred for 5 min. The

[1] Kraszkiewicz, L.; Skulski, L. *Arkivoc* **2003**, *6*, 120.

[2] Helal, C. J.; Magriotis, P. A.; Corey, E. J. *J. Am. Chem. Soc.* **1996**, *118*, 10938.

mixture was then cooled back to −78 °C and chlorotriisopropylsilane (6.4 mL, 30 mmol, 1.0 equiv) was added dropwise. The mixture was then allowed to warm to room temperature and stirred overnight. A saturated solution of ammonium chloride (40 mL) was added, and the reaction mixture was extracted with diethyl ether (2 × 60 mL). The organic layer was washed with water and brine, then dried over $MgSO_4$, filtered and concentrated under reduced pressure to obtain a colorless liquid which was further purified by Kugelrohr distillation (56–57 °C/0.25 mm of Hg) to yield **9** (7.16 g, 28.0 mmol, 92% yield) as a colorless liquid. ^{1}H NMR (400 MHz, $CDCl_3$): δ 1.08 (m, 21H, TIPS), 0.18 (s, 9H, TMS); IR ν 2959 (m), 2944 (m), 2896 (w), 2867 (m), 1464 (w), 1385 (w), 1250 (m), 996 (w), 842 (s), 764 (s), 675 (m), 660 (m). The values of the NMR spectra are in accordance with reported literature data.[2]

1-[(Triisopropylsilyl)ethynyl]-1,2-benziodoxol-3(1*H*)-one (1a)

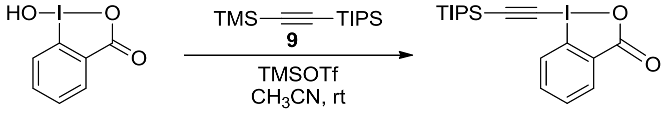

Following a reported procedure,[3] 2-iodosylbenzoic acid (**7**, 21.7 g, 82.0 mmol, 1.00 equiv) was charged in an oven-dried three-necked 1L flask equipped with a magnetic stirrer. After 3 vacuum/nitrogen cycles, anhydrous acetonitrile (500 mL) was added via canula and cooled to 0 °C. Trimethylsilyltriflate (16.4 mL, 90.0 mmol, 1.10 equiv) was added dropwise via a dropping funnel over 30 min (no temperature increase was observed). After 15 min, (trimethylsilyl)(triisopropylsilyl)acetylene (**9**, 23.0 g, 90.0 mmol, 1.10 equiv) was added via canula over 15 min (no temperature increase was observed). After 30 min, the suspension became an orange solution. After 10 min, pyridine (7.0 mL, 90 mmol, 1.1 equiv) was added via syringe. After 15 min, the reaction mixture was transferred in a one-necked 1L flask and the volume reduced under vacuum until a solid was obtained. The solid was dissolved in CH_2Cl_2 (200 mL) and transferred in a 1L separatory funnel. The organic layer was washed with 1 M HCl (200 mL) and the aqueous layer was back-extracted with CH_2Cl_2 (200 mL). The organic layers were combined, washed with a saturated solution of $NaHCO_3$ (2 x 200 mL), dried over $MgSO_4$, filtered and the solvent was evaporated under reduced pressure. Recrystallization from acetonitrile (*ca* 120 mL) afforded **1a** (30.1 g, 70.2 mmol, 86%) as colorless crystals. Mp (Dec.):

[3] Brand, J. P.; Waser, J. *Angew. Chem., Int. Ed.* **2010**, *49*, 7304.

170-176 °C; ^{1}H NMR (400 MHz, $CDCl_3$): δ 8.44 (m, 1H, Ar*H*), 8.29 (m, 1H, Ar*H*), 7.77 (m, 2H, Ar*H*), 1.16 (m, 21H, TIPS); ^{13}C NMR (100 MHz, $CDCl_3$): δ 166.4, 134.6, 132.3, 131.4, 131.4, 126.1, 115.6, 114.1, 64.6, 18.4, 11.1; IR ν 2943 (m), 2865 (m), 1716 (m), 1618 (m), 1604 (s), 1584 (m), 1557 (m), 1465 (m), 1439 (w), 1349 (m), 1291 (m), 1270 (w), 1244 (m), 1140 (m), 1016 (m), 999 (m), 883 (m), 833 (m), 742 (m), 702 (s), 636 (m). The values of the NMR spectra are in accordance with reported literature data.[3]

3. Preparation of peptides

(*S*)-Methyl 2-amino-3-(1*H*-indol-3-yl)propanoate (11)

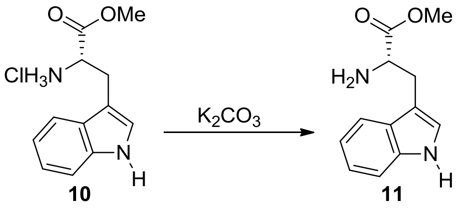

Tryptophan methyl ester hydrochloride (**10**, 22 mmol, 5.6 g, 1 equiv) was dissolved in water (25 mL). K_2CO_3 (4.56 g, 33.0 mmol, 1.5 equiv) was added and the reaction mixture was stirred for 15 min. The mixture was extracted with Et_2O (3 x 100 mL). The organic layers were combined, and dried over Na_2SO_4 and evaporated under reduced pressure to give a colorless oil. The product was then used immediately without further purification.

(*S*)-Methyl 2-((*S*)-2-(((benzyloxy)carbonyl)amino)-3-methylbutanamido)-3-(1*H*-indol-3-yl)propanoate (4a)

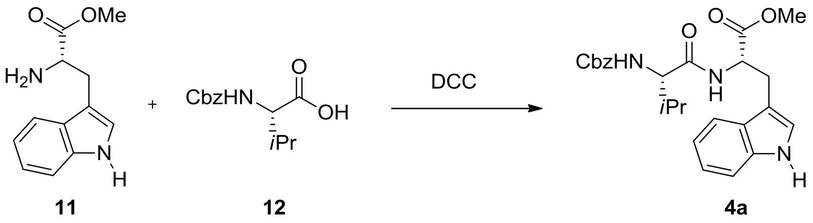

Following a modification of a reported procedure,[4] ZValOH (**12**) was dissolved in DCM (40 mL). The mixture was cooled to 0 °C, and DCC (4.13 g, 20.0 mmol, 1 equiv.) in DCM (40 mL) was added to give a white suspension. Methyl 2-amino-3-(1*H*-indol-2-yl)propanoate (**11**) (4.56 g, 20.0 mmol, 1 equiv) was added in a DCM solution (40 mL), the reaction was stirred at 0 °C for an hour, then the ice bath was removed. After stirring overnight a white suspension was obtained. The suspension was filtered through Celite, and the resulting solution was concentrated under vacuum, to give a white solid. The solid was purified by column chromatography (200 g SiO_2, hex:EtOAc 1:1), to give a white solid (~9 g).The crude product was recrystallized from MeOH (15 mL), and washed with MeOH (4 mL), to give a solid, that was dried on high vac. for several

[4]Nitta, H.; Yu, D.; Kudo, M.; Mori, A.; Inoue, S. *J. Am. Chem. Soc.* **1992**, *114*, 7969.

hours (7.9 g yield). To obtain a pure product a second recrystallization was necessary to yield peptide **4a** (4.3 g, 9.5 mmol, 48% yield) as a white solid, after drying in high vacuum.

Rf: 0.40 (hex:EtOAc 2:3).

Mp: 147-149 °C. lit.: 150-151 °C.[5]

^{1}H NMR (400 MHz, $CDCl_3$) δ 8.29 (s, 1 H, indole NH), 7.52 (d, 1 H, *J* = 7.8 Hz, ArH), 7.39-7.26 (m, 6 H, ArH), 7.18 (t, 1 H, *J* = 7.1 Hz, ArH), 7.11 (m, 1 H, ArH), 6.91 (s, 1 H, indole C3H), 6.79 (d, 1 H, *J* = 7.6 Hz, NH), 5.52 (d, 1 H, *J* = 9.1 Hz, NH), 5.06 (m, 1 H, CH), 5.00-4.85 (m, 2 H, Cbz CH_2), 4.25 (dd, 1 H, *J* = 8.8, 6.0 Hz, CH), 3.67 (s, 3 H, OMe), 3.32 (dd, 1 H, *J* = 14.8, 5.5 Hz, CH_2), 3.28 (dd, 1 H, *J* = 15.0, 5.2 Hz, CH_2), 2.10 (m, 1 H, Val CH), 0.96 (d, 3 H, *J* = 6.7 Hz, Val Me), 0.90 (d, 3 H, *J* = 6.7 Hz, Val Me).

^{13}C NMR (101 MHz, $CDCl_3$) δ 172.1, 171.4, 156.5, 136.3, 136.1, 128.6, 128.1, 127.4, 123.3, 122.2, 119.6, 118.4, 111.4, 109.3, 67.0, 60.0, 52.8, 52.4, 31.5, 27.6, 19.2, 17.6.

IR 3407 (w), 3401 (w), 3315 (w), 3314 (w), 3313 (w), 2964 (w), 1711 (m), 1658 (m), 1516 (m), 1455 (w), 1441 (w), 1344 (w), 1266 (m), 1217 (m), 1100 (w), 1030 (w), 736 (s).

The ^{13}C NMR data fits the reported data, significant shifts from the reported values were observed for the ^{1}H NMR data.[5]

(*S*)-methyl 2-(2-(((benzyloxy)carbonyl)amino)acetamido)-3-(1*H*-indol-3-yl)propanoate (4b)

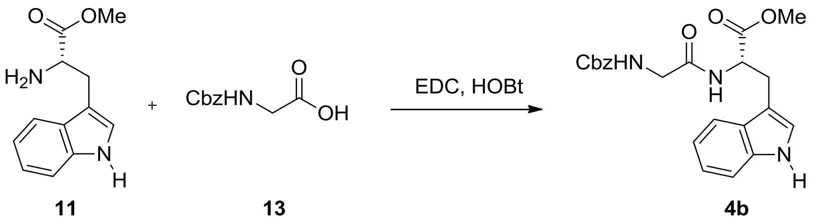

ZGlyOH (**13**) (1.05 g, 5.00 mmol, 1.06 equiv) and tryptophan ester **11** (1.20 g, 4.71 mmol, 1 equiv) were dissolved in DCM (50 mL). Diisopropylethyl amine (2.2 mL, 12 mmol, 2.5 equiv) and HOBt (0.766 g, 5.00 mmol, 1.06 equiv) were added. The reaction mixture was cooled to 0 °C, and EDC (0.959 g, 5.00 mmol, 1.06 equiv) was added. The reaction mixture was allowed to warm to room temperature and stirred overnight. The solvent was evaporated under reduced

[5]Sperry, J.; Moody, C. J. *Tetrahedron* **2010**, *66*, 6483.

pressure and the resulting thick oil dissolved in ethyl acetate (100 mL). The organic layer was washed with 5% $KHSO_4$ (2 × 20 mL), conc. $NaHCO_3$ (50 mL) and brine (50 mL), dried over $MgSO_4$ and the solvent was evaporated under reduced pressure. The crude oil was purified by column chromatography (100 g SiO_2, hex/EtOAc 1:1) to give peptide **4b** (1.85 g, 4.52 mmol, 96% yield) as a white amorphous solid, after drying in high vacuo. Compound **4b** still contained a small amount of impurities (<10%), which were not removed by column chromatography.

^{1}H NMR (400 MHz, $CDCl_3$) δ 8.19 (br s, 1 H, indole NH), 7.47 (d, 1 H, *J* = 7.8 Hz, ArH), 7.41-7.23 (m, 6 H, ArH), 7.15 (m, 1 H, ArH), 7.09 (m, 1 H, ArH), 6.87 (s, 1 H, indole C3H), 6.63 (d, 1 H, *J* = 7.7 Hz, NH), 5.44 (m, 1 H, NH), 5.09 (d, 1H, *J* = 12.3 Hz, Cbz CH_2), 5.05 (d, 1H, *J* = 12.2 Hz, Cbz CH_2), 4.91 (m, 1 H, CH), 3.84-3.72 (m, 2 H, CH_2), 3.65 (s, 3 H, OMe), 3.29 (m, 2 H, Trp CH_2).

^{13}C NMR (101 MHz, $CDCl_3$) δ 172.1, 168.7, 156.5, 136.1, 136.0, 128.5, 128.2, 128.1, 127.3, 123.1, 122.1, 119.6, 118.2, 111.3, 109.3, 67.1, 52.7, 52.5, 44.3, 27.4.

IR 3331 (w), 3062 (w), 2952 (w), 1713 (s), 1668 (s), 1520 (m), 1440 (m), 1345 (m), 1217 (s), 911 (m), 735 (s).

HRMS (ESI) calcd. for $C_{22}H_{24}N_3O_5^+$ $[M+H]^+$ 410.1710; found 410.1731.

(*S*)-Methyl 2-((*S*)-2-(((benzyloxy)carbonyl)amino)-3-phenylpropanamido)-3-(1*H*-indol-3-yl)propanoate (4c)

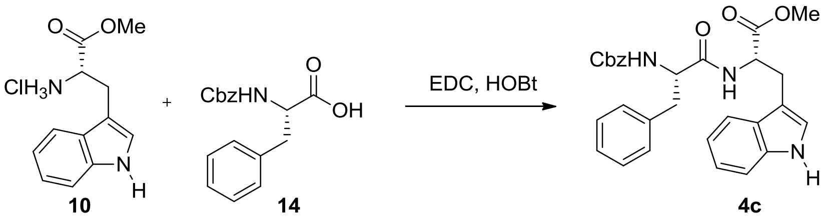

In a 250 mL round-bottomed flask, ZPheOH (**14**) (1.49 g, 5.00 mmol, 1 equiv) was dissolved in dry DCM (50 mL), then 3-(1*H*-indol-3-yl)-1-methoxy-1-oxopropan-2-aminium chloride (**10**, 1.27 g, 5.00 mmol, 1 equiv) was added to give a white suspension. DIPEA (2.18 mL, 12.5 mmol, 2.5 equiv) was added and the mixture became a colorless solution. HOBT (0.919 g, 6.00 mmol, 1.2 equiv) was added, and the mixture was cooled in an ice-bath. Then EDC (1.05 g, 5.50 mmol, 1.1 equiv) was added and the reaction mixture was stirred overnight while it was allowed to warm up

to room temperature. The solvent was evaporated under reduced pressure, and the residue was dissolved in EtOAc (100 mL). The organic layer was extracted with 5% $KHSO_4$ (2x20 mL), conc. $NaHCO_3$ (20 mL), brine (50 mL) and dried over $MgSO_4$. The solvent was evaporated under reduced pressure, and the resulting oil was purified via column chromatography (SiO_2, pentane/EtOAc, 1:1), to give peptide **4c** (2.30 g, 4.60 mmol, 92% yield) as a white solid.

Rf= 0.45 (hex:EtOAc 1:1).

Mp: 130-134 °C.lit 119-120 °C.[6]

^{1}H NMR (400 MHz, $CDCl_3$) δ 8.13 (s, 1 H, indole NH), 7.40 (d, 1 H, *J* = 7.9 Hz, ArH), 7.25 (m, 12 H, ArH), 7.06 (t, 1 H, *J* = 7.1, ArH), 6.81 (s, 1 H, indole C3 H), 6.52 (d, 1 H, *J* = 6.8 Hz, NH), 5.34 (d, 1 H, *J* = 7.7 Hz, NH), 4.99 (q, 2 H, *J* = 1.2 Hz), 4.88 (dt, 1 H, *J* = 7.7, 5.5 Hz, CH), 4.54 (m, 1 H, CH), 3.66 (s, 3 H, OMe), 3.27 (dd, 1 H, *J* = 15.1, 5.5 Hz, CH_2), 3.26 (dd, 1 H, *J* = 14.9, 5.4 Hz, CH_2), 3.02 (m, 2 H, CH_2).

^{13}C NMR (101 MHz, $CDCl_3$) δ 171.8, 170.7, 155.9, 136.3, 136.1, 129.4, 128.7, 128.6, 128.2, 128.0, 127.4, 127.0, 123.1, 122.2, 119.7, 118.4, 111.4, 109.5, 67.0, 56.0, 53.0, 52.4, 38.5, 27.6. One aromatic C is not resolved.

IR 3409 (w), 3325 (w), 3324 (w), 3323 (w), 3061 (w), 3060 (w), 3033 (w), 2952 (w), 1737 (m), 1710 (m), 1660 (m), 1659 (m), 1516 (m), 1455 (w), 1440 (w), 1342 (w), 1261 (m), 1214 (m), 1183 (w), 1182 (w), 1109 (w), 1108 (w), 1048 (w), 1029 (w), 737 (s).

Significant shifts from the reported values were observed for the ^{1}H NMR data.[6]

(*S*)-methyl 2-((*S*)-2-(((benzyloxy)carbonyl)amino)-3-(4-hydroxyphenyl)propanamido)-3-(1*H*-indol-3-yl)propanoate (4d)

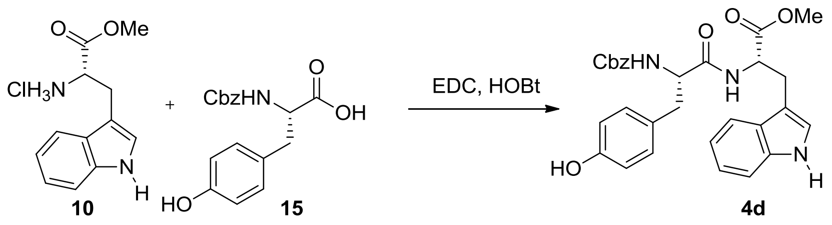

[6]Kolesinska, B.; Kaminski, Z. J. *Org. Lett.* **2009**, *11*, 765.

Dry DCM (50 mL) was filled into a 100 mL round-bottomed flask. The hydrochloride salt 3-(1*H*-indol-3-yl)-1-methoxy-1-oxopropan-2-aminium chloride (**10**) (1.27 g, 5.00 mmol, 1 equiv) was suspended with stirring. 2-(((benzyloxy)carbonyl)amino)-3-(4-hydroxyphenyl)propanoic acid (**15**) (1.58 g, 5.00 mmol, 1 equiv) was added. DIPEA (2.18 mL, 12.5 mmol, 2.5 equiv) was added, while the suspension cleared out to give a colorless solution. HOBT (0.766 g, 5.00 mmol, 1 equiv.) was added. The reaction mixture was cooled in an ice bath and EDC (0.959 g, 5.00 mmol, 1 equiv.) was added. The reaction mixture was stirred overnight, while it was allowed to warm up to room temperature. The residue was dissolved in EtOAc (100 mL), washed with 5% $KHSO_4$ (2 x 20 mL), then with conc. $NaHCO_3$ (20 mL) and brine (50 mL). The organic layer was dried over $MgSO_4$.The solvent was evaporated under reduced pressure and the residue was purified by column chromatography (SiO_2, hex/EtOAc, 3:2) to give peptide **4d** (1.86 g, 3.61 mmol, 72% yield) as a white solid.

Rf = 0.35 (hex:EtOAc 2:3).

Mp: 79-85 °C lit. 113-116 °C.[6]

^{1}H NMR (400 MHz, $CDCl_3$) δ 8.23 (s, 1 H, indole NH), 7.44-7.26 (m, 8 H, ArH), 7.16 (m, 1 H, ArH), 7.07 (m, 1 H, ArH), 6.91 (d, 2 H, *J* = 7.5 Hz, ArH), 6.76 (s, 1 H, ArH), 6.64-6.47 (m, 2 H, ArH), 6.37 (d, 1 H, *J* = 7.2 Hz, NH), 5.45 (d, 1 H, *J* = 7.9 Hz, NH), 5.09-4.99 (m, 2 H, Cbz CH_2), 4.83 (m, 1 H, CH), 4.38 (m, 1 H, CH), 3.58 (s, 3 H, OMe), 3.23 (m, 2 H, CH_2), 2.88 (m, 2 H, CH_2).

^{13}C NMR (101 MHz, $CDCl_3$) δ 172.0, 171.5, 156.2, 155.3, 136.1, 136.0, 130.4, 128.6, 128.3, 128.0, 127.5, 127.3, 123.4, 122.1, 119.6, 118.3, 115.7, 111.5, 109.0, 67.2, 56.3, 53.1, 52.5, 37.9, 27.5.

IR 3399 (w), 3384 (w), 3340 (w), 3325 (w), 3057 (w), 3037 (w), 2953 (w), 1707 (m), 1659 (m), 1616 (w), 1597 (w), 1515 (s), 1454 (m), 1453 (m), 1441 (m), 1342 (w), 1260 (m), 1220 (s), 1178 (m), 1130 (w), 1105 (w), 1049 (w), 1027 (w), 830 (w), 811 (w), 737 (s).

Significant shifts from the reported values were observed for the ^{1}H NMR data.[6]

(*S*)-Methyl 2-((*S*)-2-(((benzyloxy)carbonyl)amino)-3-hydroxypropanamido)-3-(1*H*-indol-3-yl)propanoate (4e)

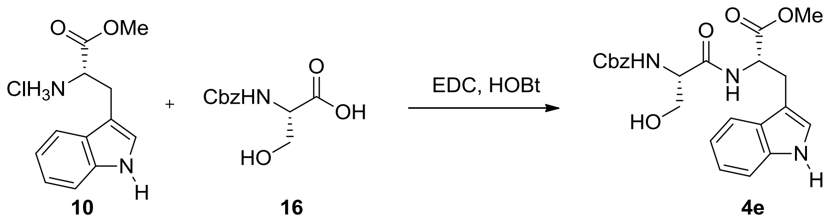

3-(1*H*-indol-3-yl)-1-methoxy-1-oxopropan-2-aminium chloride (**10**, 2.55 g, 10.0 mmol, 1 equiv) was suspended in 100 mL of dry DCM, then 2-(((benzyloxy)carbonyl)amino)-3-hydroxypropanoic acid (**16**) (2.39 g, 10.0 mmol, 1 equiv) was added. *N*-ethyl-*N*-isopropylpropan-2-amine (4.17 mL, 25.0 mmol, 2.5 equiv) was added and the white solid dissolved. HOBT (1.83 g, 12.0 mmol, 1.2 equiv) was added. The reaction mixture was cooled in an ice bath, then EDC (2.11 g, 11.0 mmol, 1.1 equiv) was added. The resulting solution was stirred overnight while it was allowed to warm to rt. The solvent was evaporated and the resulting viscous oil was dissolved in a mixture of EtOAc (200 mL) and DCM (10 mL). The organic layer was washed with 5% $KHSO_4$ (2 x 20 mL), conc. $NaHCO_3$ (20 mL), conc. NaCl (50 mL) and dried over $MgSO_4$. The solvent was evaporated under reduced pressure. Et_2O (50 mL) was added into the flask, and evaporated to get a foam, which was dried under vacuum for 1 h (3.6 g, white solid). The crude product was purified via column chromatogaraphy (SiO_2, hexane/ethyl acetate 2:8), to get peptide **4e** (2.54 g, 5.78 mmol, 58% yield) as a white solid.

Rf: 0.25 (Hex/EtOAc 1/4).

Mp: 60-62 °C. lit. 99-100 °C.[7]

^{1}H NMR (400 MHz, $CDCl_3$) δ 8.31 (s, 1 H, indole NH), 7.51 (d, 1 H, *J* = 7.8 Hz, ArH), 7.40-7.24 (m, 5 H, ArH), 7.21-7.06 (m, 3 H, ArH and OH), 6.93 (d, 1 H, *J* = 2.3 Hz, ArH), 5.87 (d, 1 H, *J* = 7.7 Hz, NH), 5.05 (m, 2 H, Cbz CH_2), 4.90 (m, 1 H, CH), 4.25 (br s, 1 H, NH), 3.90 (m, 1 H, CH), 3.70 (s, 3 H, OMe), 3.58 (m, 1 H), 3.36-3.20 (m, 3 H).

[7]Ranganathan, D.; Vaish, N. K.; Shah, K. *J. Am. Chem. Soc.* **1994**, *116*, 6545.

^{13}C NMR (101 MHz, $CDCl_3$) δ 172.4, 170.7, 156.5, 136.1, 128.6, 128.3, 128.1, 127.3, 123.2, 122.2, 119.6, 118.3, 111.5, 109.3, 67.2, 62.9, 55.6, 53.0, 52.7, 27.2. One aromatic C is not resolved.

IR 3401 (w), 3400 (w), 3379 (w), 3378 (w), 3377 (w), 3337 (w), 3332 (w), 3331 (w), 3330 (w), 3059 (w), 3058 (w), 2953 (w), 1721 (m), 1714 (m), 1713 (m), 1662 (m), 1517 (m), 1455 (w), 1440 (w), 1403 (w), 1391 (w), 1342 (w), 1264 (m), 1216 (m), 1183 (w), 1060 (m), 1059 (m), 1028 (w), 1027 (w), 736 (s).

Significant shifts from the reported values were observed for the ^1H NMR data.[7]

(*S*)-Benzyl 2-(((*S*)-3-(1*H*-indol-3-yl)-1-methoxy-1-oxopropan-2-yl)carbamoyl)pyrrolidine-1-carboxylate (4f)

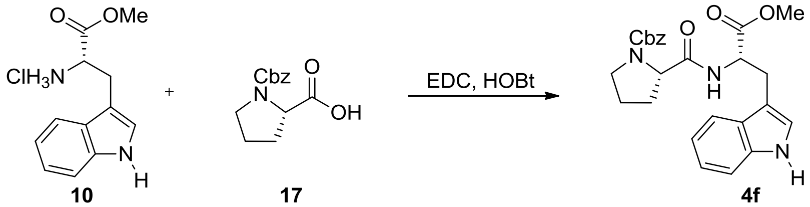

3-(1*H*-indol-3-yl)-1-methoxy-1-oxopropan-2-aminium chloride (**10**, 1.27 g, 5.00 mmol, 1 equiv) was suspended in 50 mL of dry DCM, then amino acid **17** (1.25 g, 5.00 mmol, 1 equiv) was added. *N*-Ethyl-*N*-isopropylpropan-2-amine (2.1 mL, 12 mmol, 2.5 equiv) was added and the white solid dissolved. HOBT (0.92 g, 6.0 mmol, 1.2 equiv) was added. The reaction mixture was cooled in an ice bath, then EDC (1.05 g, 5.50 mmol, 1.1 equiv) was added. The resulting solution was stirred overnight while it was allowed to warm to rt. The solvent was evaporated and the resulting viscous oil was dissolved in EtOAc (100 mL). The organic layer was washed with 5% $KHSO_4$ (2 × 20 mL), conc. $NaHCO_3$ (20 mL), conc. NaCl (50 mL) and dried over $MgSO_4$. The solvent was evaporated under reduced pressure. The crude product was purified via column chromatography (SiO_2, hexane/ethyl acetate 2:8), to get peptide **4f** (1.98 g, 4.40 mmol, 88% yield) as an oil.

Rf: 0.45 (Hex/EtOAc 1/4).

^1H NMR (400 MHz, $CDCl_3$) δ 8.30-7.96 (br s, 1 H, indole NH), 7.54-7.46 (m, 1 H, ArH), 7.37-7.27 (m, 5 H, ArH), 7.22-6.94 (m, 3 H, ArH), 6.83 (s, 0.5 H, rotamer 1), 6.37 (s, 0.5 H, rotamer

2), 5.21-4.96 (m, 2 H, Cbz CH_2), 4.93-4.83 (m, 1 H), 4.41-4.16 (m, 1 H), 3.76-3.56 (m, 3 H), 3.53-3.12 (m, 4 H), 2.42-1.29 (m, 4 H). Broadening of all peaks was observed due to rotamers.

IR 3321 (w), 3058 (w), 2953 (w), 1742 (m), 1673 (s), 1519 (m), 1421 (s), 1357 (s), 1211 (s), 1119 (m), 741 (s).

^{1}H NMR data is in agreement with the reported data.[8]

(*S*)-Methyl 2-((*S*)-2-(((benzyloxy)carbonyl)amino)-3-(1*H*-indol-3-yl)propanamido)-3-methylbutanoate (4g)

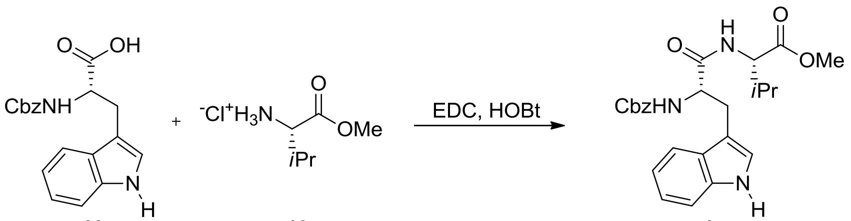

DCM (100 mL) was added to 2-(((benzyloxy)carbonyl)amino)-3-(1*H*-indol-3-yl)propanoic acid (**18**, 3.38 g, 10.0 mmol, 1 equiv.) and (*S*)-valine methylester hydrochloride (**19**) (1.536 g, 10.00 mmol, 1 equiv). Then DIPEA (4.37 mL, 25.0 mmol, 2.5 equiv) was added, and the reaction mixture became a colorless solution. HOBT (1.53 g, 10.0 mmol, 1 equiv) was added. The reaction mixture was cooled in an ice-bath, then EDC (1.92 g, 10.0 mmol, 1 equiv) was added. The resulting solution was stirred overnight while it was allowed to warm to rt and stirred over the weekend, shielded from light. The solvent was evaporated under reduced pressure, and the resulting thick oil was taken up into EtOAc (450 mL). The solution was washed with 5% $KHSO_4$ (2 × 50 mL), conc. $NaHCO_3$ (50 mL), and brine (50 mL). The organic layer was dried over $MgSO_4$, and the solvent was evaporated under reduced pressure. The crude oil was purified by column chromatography (100 g SiO_2, hex/EtOAc 1:1), to give peptide **4g** (3.90 g, 8.64 mmol, 86% yield), as a white solid.

Mp: 60-66 °C.

^{1}H NMR (400 MHz, $CDCl_3$) δ 8.10 (s, 1 H, indole NH), 7.71 (d, 1 H, *J* = 7.1 Hz, ArH), 7.41-7.27 (m, 6 H, ArH), 7.22 (m, 1 H, ArH), 7.11 (m, 2 H, ArH), 6.16 (d, 1 H, *J* = 7.7 Hz, NH), 5.55 (d, 1

[8]Duan, J.; Sun, Y.; Chen, H.; Qiu, G.; Zhou, H.; Tang, T.; Deng, Z.; Hong, X. *J. Org. Chem.*, **2013**, *78*, 7013.

H, J = 6.7 Hz, NH), 5.15 (s, 2 H, Cbz CH_2), 4.55 (m, 1 H, CH), 4.41 (dd, 1 H, J = 8.4, 5.0 Hz, CH), 3.66 (s, 3 H, OMe), 3.35 (dd, 1 H, J = 14.4, 4.8 Hz, CH_2), 3.20 (dd, 1 H, J = 14.5, 8.0 Hz, CH_2), 2.03 (m, 1 H, Val CH), 0.78 (d, 3 H, J = 6.8 Hz, Val CH_3), 0.74 (d, 3 H, J = 6.9 Hz, Val CH_3).

^{13}C NMR (101 MHz, $CDCl_3$) δ 171.7, 171.1, 156.0, 136.3, 128.6, 128.2, 128.1, 127.4, 123.4, 122.4, 119.9, 118.9, 111.2, 110.5, 67.1, 57.4, 55.6, 52.1, 31.2, 28.6, 18.7, 17.8. One aromatic carbon is not resolved.

IR 3323 (w), 3059 (w), 2964 (w), 1708 (s), 1663 (s), 1532 (s), 1455 (m), 1344 (m), 1268 (s), 1219 (s), 1146 (m), 1024 (m), 742 (s).

HRMS (ESI) calcd for $C_{25}H_{30}N_3O_5^+$ $[M+H]^+$ 452.2180; found 452.2214.

(5*S*, 8*S*, 11*S*)-Methyl 8-((1*H*-indol-3-yl)methyl)-5,11-diisopropyl-3,6,9-trioxo-1-phenyl-2-oxa-4,7,10-triazadodecan-12-oate (4h)

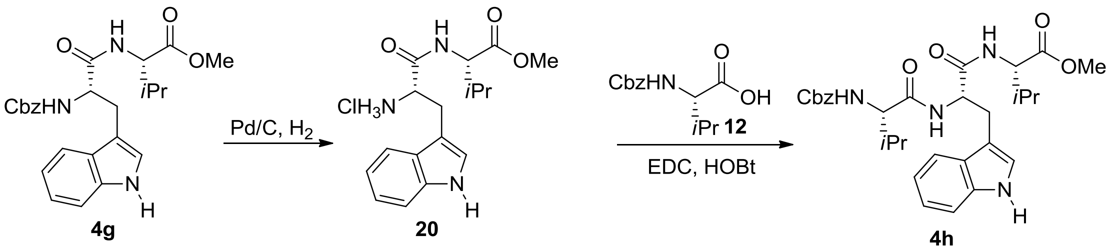

The starting peptide **4g** (2.25 g, 5.00 mmol, 1 equiv) and Pd/C (dry, 10% W%, Degussa E105CA/W, 532 mg, 0.500 mmol, 0.1 equiv) were added into a dry 25 mL round-bottomed flask. The atmosphere was changed to N_2 by evacuating and refilling with nitrogen 3 times. 50 mL of dry MeOH was added. H_2 was bubbled through the solution with strong stirring for 20 minutes, at which time TLC did not show the starting material (Rf(SM) = 0.30 Hex/EtOAc 1:1), but only one spot that is not moving even in hex/EtOAc 2:3. Celite was added, then the reaction mixture was filtered through a plug of celite. The solvent was evaporated to give a colorless oil. The residue was dissolved in Et_2O (50 mL) to give an opaque solution. 3 N HCl in CPME (2 mL) was added with stirring to form a slowly solidifying oil. The solvent was evaporated under reduced pressure, and Et_2O (20 mL) was added, and evaporated under reduced pressure, to give peptide **20** (1.70 g, 4.80 mmol, 96% yield) as a white solid, which was directly used without further purification.

(*S*)-2-(((Benzyloxy)carbonyl)amino)-3-methylbutanoic acid (**12**) (263 mg, 1.04 mmol, 1.1 equiv) and peptide **20** (336 mg, 0.950 mmol, 1 equiv) were stirred in DCM (10 mL) to give a white suspension. DIPEA (0.415 mL, 2.37 mmol, 2.5 equiv) was added in one portion to give a colorless solution. HOBT (306 mg, 1.99 mmol, 2.1 equiv) was added, then the solution was cooled to 0 °C in an ice-bath. After 10 min., EDC (364 mg, 1.90 mmol, 2 equiv) was added. The resulting solution was stirred overnight, while it was allowed to warm up to rt. A suspension is formed. The solvent was removed under reduced pressure, and the crude was dissolved in EtOAc (100 mL). The organic layer was washed with 5% $KHSO_4$ (2 × 20 mL), conc. $NaHCO_3$ (20 mL), and brine (50 mL). The solution was dried over $MgSO_4$, and the solvent was evaporated under reduced pressure. The product was purified by column chromatography (SiO_2, hex/EtOAc 2:1) to give peptide **4h** (86.0 mg, 0.156 mmol, 16% yield) as an amorphous solid.

Rf = 0.40 (hex/EtOAc 3/2).

^{1}H NMR (400 MHz, DMSO) δ 10.80 (s, 1 H, indole NH), 8.15 (d, 1 H, *J* = 8.1 Hz, ArH or NH), 8.01 (d, 1 H, *J* = 8.1 Hz, ArH or NH), 7.56 (d, 1 H, *J* = 7.8 Hz, Ar H or NH), 7.38-7.20 (m, 5 H, ArH), 7.30 (d, *J* = 7.9 Hz, ArH or NH), 7.22, (d, 1 H, *J* = 9.0 Hz, ArH or NH), 7.14 (d, 1 H, *J* = 1.7 Hz, ArH), 7.04 (t, 1 H, *J* = 7.1 Hz, ArH), 6.96 (t, 1 H, *J* = 7.3 Hz, ArH), 5.04 (d, 1 H, *J* = 12.7 Hz, Cbz CH_2), 5.00 (d, 1 H, *J* = 12.6 Hz, Cbz CH_2), 4.69 (dd, 1 H, *J* = 13.8, 7.9 Hz, CH), 4.16 (dd, 1 H, *J* = 7.9, 6.6 Hz, CH), 3.86 (dd, 1 H, *J* = 8.7, 7.2 Hz, CH), 3.59 (s, 3 H, OMe), 3.08 (dd, 1 H, *J* = 14.8, 5.6 Hz, CH_2-tryptophan), 2.94 (dd, 1 H, *J* = 14.8, 8.3 Hz, CH_2-tryptophan), 2.04-1.95 (m, 1 H, Val CH), 1.89 (m, 1 H, Val CH), 0.85 (d, 3 H, *J* = 6.8 Hz, Val Me), 0.84 (d, 3 H, *J* = 6.8 Hz, Val Me), 0.76 (d, 6 H, *J* = 6.7 Hz, Val Me).

^{13}C NMR (101 MHz, DMSO) δ 171.7, 171.7, 170.9, 156.0, 137.0, 136.0, 128.3, 127.7, 127.6, 127.3, 123.5, 120.8, 118.4, 118.1, 111.1, 109.8, 65.4, 60.2, 57.4, 54.9, 52.8, 51.6, 30.4, 29.9, 27.7, 19.1, 18.9, 18.2.

4. Alkynylation of peptides

4.1 General procedure

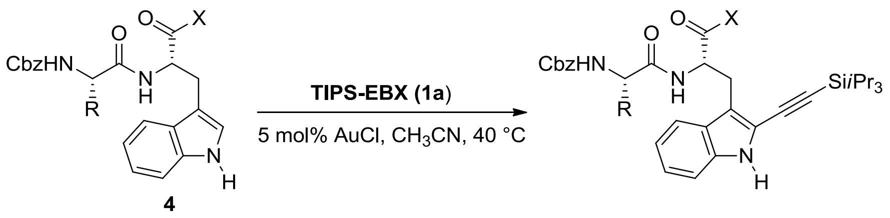

The starting peptide **4** (0.20 mmol, 1 equiv) and TIPS-EBX (**1a**, 0.240 mmol, 103 mg, 1.2 equiv) were added into a 5 mL test-tube equipped with a stirring bar. Acetonitrile (2 mL) was added, then the reaction mixture was stirred at 40 °C for 2 min and gold(I) chloride (2.3 mg, 10 µmol, 0.05 equiv) was added in one portion. Afterwards, the reaction tube was sealed and stirred for 24 h at 40 °C. The mixture was diluted with EtOAc (50 mL), and the organic layer was washed with a mixture of water (2.5 mL) and conc. $NaHCO_3$ solution (2.5 mL), and then with brine (20 mL), and dried over $MgSO_4$. The solvent was evaporated under reduced pressure and the resulting yellow oil was purified by column chromatography (SiO_2, hexane/EtOAc 3:1 to 2:3). The product was dried under reduced pressure, and washed into a vial with Et_2O. The solvent was evaporated under vacuum and dried under high vacuum (ca. 10^{-2} mbar) for several hours.

4.2. Scope of the Alkynylation of Peptides

(*S*)-methyl 2-((*S*)-2-(((benzyloxy)carbonyl)amino)-3-methylbutanamido)-3-(2-((triisopropylsilyl)ethynyl)-1*H*-indol-3-yl)propanoate (5a)

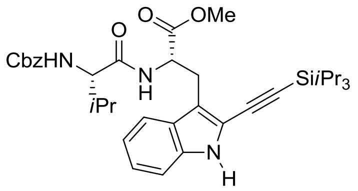

5a

Starting from peptide **4a** (90 mg, 0.20 mmol) alkyne **5a** (98.1 mg, 0.155 mmol, 78% yield) was obtained as a white solid.

Rf: 0.80 (hex:EtOAc 2:3).

Mp: 70-73 °C.

^{1}H NMR (400 MHz, $CDCl_3$) δ 8.16 (s, 1 H, indole NH), 7.59 (d, 1 H, *J* = 7.9 Hz, ArH), 7.40-7.21 (m, 7 H, ArH), 7.13 (t, 1 H, *J* = 7.5 Hz, ArH), 6.25 (d, 1 H, *J* = 6.9 Hz, NH), 5.32 (d, 1 H, *J* = 8.6 Hz, NH), 5.08 (s, 2 H, Cbz CH_2), 4.83 (dd, 1 H, *J* = 7.2, 7.2 Hz, CH), 4.02 (dd, 1 H, *J* = 8.4, 5.3 Hz, CH), 3.69 (s, 3 H, OMe), 3.37 (dd, 1 H, *J* = 14.3, 6.1 Hz, Trp CH_2), 3.31 (dd, 1 H, *J* = 14.2, 7.8 Hz, Trp CH_2), 2.06 (m, 1 H, Val CH), 1.17 (m, 21 H, TIPS), 0.92 (d, 3 H, *J* = 6.7 Hz, Val Me), 0.81 (d, 3 H, *J* = 6.7 Hz, Val Me).

^{13}C NMR (101 MHz, $CDCl_3$) δ 171.9, 170.7, 156.1, 136.3, 135.6, 128.5, 128.1, 128.1, 126.7, 124.2, 120.7, 119.1, 118.1, 116.7, 111.1, 98.6, 97.4, 66.9, 59.8, 53.1, 52.5, 31.7, 19.0, 18.7, 17.3, 11.3.

IR 3411 (w), 3410 (w), 3407 (w), 3406 (w), 3318 (w), 3317 (w), 3316 (w), 3062 (w), 2945 (m), 2892 (w), 2866 (m), 2146 (w), 1729 (s), 1713 (s), 1663 (s), 1511 (s), 1505 (s), 1460 (m), 1440 (m), 1369 (m), 1348 (m), 1279 (m), 1278 (m), 1217 (s), 1180 (m), 1025 (m), 998 (m), 883 (m), 741 (s), 722 (s).

HRMS (ESI) calcd for $C_{36}H_{50}N_3O_5Si^+$ $[M+H]^+$ 632.3514; found 632.3502.

(*S*)-methyl 2-(2-(((benzyloxy)carbonyl)amino)acetamido)-3-(2-((triisopropylsilyl)ethynyl)-1*H*-indol-3-yl)propanoate (5b)

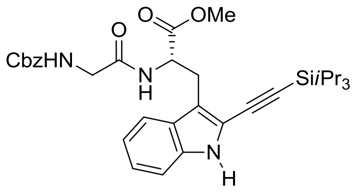

5b

Starting from peptide **4b** (82 mg, 0.20 mmol) alkyne **5b** (77 mg, 0.13 mmol, 66% yield) was obtained as an oil.

^{1}H NMR (400 MHz, $CDCl_3$) δ 8.16 (s, 1 H, indole NH), 7.54 (d, 1 H, *J* = 7.9 Hz, ArH), 7.41-7.16 (m, 7 H, ArH), 7.13 (m, 1 H, ArH), 6.35 (d, 1 H, *J* = 7.2 Hz, NH), 5.30 (br s, 1 H, NH), 5.30 (s, 2 H, Cbz CH_2), 4.88 (dd, 1 H, *J* = 6.7, 6.7 Hz, CH), 3.84 (m, 2 H, CH_2), 3.69 (s, 3 H, OMe), 3.36 (m, 2H, Trp CH_2), 1.21-1.08 (m, 21 H, TIPS).

^{13}C NMR (101 MHz, $CDCl_3$) δ 171.9, 168.2, 156.2, 136.3, 135.6, 128.6, 128.2, 128.1, 126.8, 124.2, 120.6, 119.0, 118.2, 116.6, 111.1, 98.5, 97.6, 67.1, 53.0, 52.6, 44.2, 28.0, 18.7, 11.3.

IR 3320 (w), 3062 (w), 2948 (m), 2867 (w), 2146 (w), 1730 (s), 1674 (s), 1516 (s), 1446 (m), 1352 (m), 1221 (s), 1051 (w), 997 (m), 883 (w), 741 (s).

HRMS (ESI) calcd for $C_{33}H_{44}N_3O_5Si^+$ $[M+H]^+$ 590.3045; found 590.3041.

(*S*)-Methyl 2-((*S*)-2-(((benzyloxy)carbonyl)amino)-3-phenylpropanamido)-3-(2-((triisopropylsilyl)ethynyl)-1*H*-indol-3-yl)propanoate (5c)

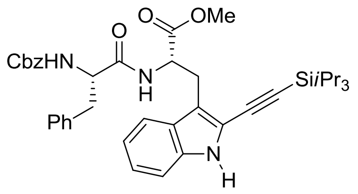

5c

Starting from peptide **4c** (100 mg, 0.200 mmol) alkyne **5c** (86.5 mg, 0.127 mmol, 64% yield) was obtained as a white solid.

Rf: 0.70 (hex:EtOAc 2:3).

Mp: 65-71 °C.

^{1}H NMR (400 MHz, $CDCl_3$) δ 8.09 (s, 1 H, indole NH), 7.45-7.05 (m, 14 H, ArH), 6.21 (d, 1 H, *J* = 7.2 Hz, NH), 5.24 (d, 1 H, *J* = 7.2 Hz, NH), 5.05 (s, 2 H, Cbz CH_2), 4.82 (dd, 1 H, *J* = 6.8, 6.8 Hz, CH), 4.37 (m, 1 H, CH), 3.67 (s, 3 H, OMe), 3.32-3.24 (m, 2 H), 3.00 (dd, 1 H, *J* = 13.7, 7.0 Hz, CH_2), 2.93 (dd, 1 H, *J* = 13.2, 5.6 Hz, CH_2), 1.32-1.07 (m, 21 H, TIPS).

^{13}C NMR (101 MHz, $CDCl_3$) δ 171.6, 170.2, 156.0, 136.3, 135.5, 129.4, 128.5, 128.5, 128.1, 128.0, 127.0, 126.9, 124.1, 120.6, 119.1, 118.1, 116.7, 111.1, 98.5, 97.3, 66.8, 56.0, 53.0, 52.5, 38.7, 28.2, 18.7, 11.3. One aromatic carbon did not resolve

IR 3408 (w), 3407 (w), 3406 (w), 3405 (w), 3399 (w), 3399 (w), 3398 (w), 3335 (w), 3323 (w), 3320 (w), 3062 (w), 3061 (w), 3032 (w), 2945 (w), 2892 (w), 2865 (w), 2146 (w), 1727 (m), 1668 (m), 1499 (m), 1455 (m), 1454 (m), 1439 (m), 1366 (w), 1347 (w), 1245 (m), 1244 (m), 1215 (m), 1181 (w), 1180 (w), 1120 (w), 1047 (w), 1032 (w), 1031 (w), 998 (w), 883 (w), 739 (s).

HRMS (ESI) calcd. for $C_{40}H_{50}N_3O_5Si^+$ $[M+H]^+$ 680.3514; found 680.3501.

(*S*)-Methyl 2-((*S*)-2-(((benzyloxy)carbonyl)amino)-3-(4-hydroxyphenyl)propanamido)-3-(2-((triisopropylsilyl)ethynyl)-1*H*-indol-3-yl)propanoate (5d)

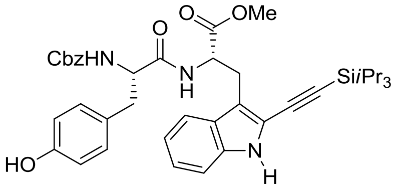

5d

Starting from peptide **4d** (103 mg, 0.200 mmol) alkyne **5d** (93.1 mg, 0.134 mmol, 67% yield) was obtained as a slightly yellow solid.

Rf: 0.65 (hex:EtOAc 2:3).

Mp: 85-89 °C.

^{1}H NMR (400 MHz, $CDCl_3$) δ 8.16 (s, 1 H, indole NH), 7.44-7.19 (m, 8 H, ArH), 7.08 (t, 1 H, *J* = 7.4 Hz, ArH), 6.96 (d, 2 H, *J* = 7.5 Hz, ArH), 6.57 (d, 2 H, *J* = 7.8 Hz, ArH), 6.13 (d, 1 H, *J* = 7.1 Hz, NH), 5.32 (m, 1 H, NH), 5.07 (s, 2 H, Cbz CH_2), 4.80 (dd, 1 H, *J* = 6.6, 6.6 Hz, CH), 4.31 (m, 1 H, CH), 3.68 (s, 3 H, OMe), 3.29 (d, 2 H, *J* = 6.3 Hz, CH_2), 2.87 (br d, 2 H, *J* = 6.3 Hz, CH_2), 1.32-1.05 (m, 21 H, TIPS).

^{13}C NMR (101 MHz, $CDCl_3$) δ 171.6, 170.4, 155.6, 154.5, 136.3, 135.5, 130.6, 128.5, 128.2, 128.0, 126.9, 124.1, 120.6, 119.0, 118.2, 116.4, 115.4, 111.1, 98.6, 97.2, 66.9, 56.3, 53.0, 52.5, 38.1, 27.9, 18.7, 11.3. One aromatic carbon is not resolved.

IR 3388 (m), 3387 (m), 3384 (m), 3383 (m), 3382 (m), 3381 (m), 3380 (m), 3379 (m), 3345 (m), 3345 (m), 3344 (m), 3343 (m), 3342 (m), 3341 (m), 3340 (m), 2947 (m), 2946 (m), 2866 (w), 2865 (w), 2147 (w), 1723 (s), 1670 (s), 1669 (s), 1613 (w), 1516 (s), 1515 (s), 1451 (m), 1354 (m), 1353 (m), 1352 (m), 1228 (s), 1117 (w), 1116 (w), 1054 (w), 1053 (w), 1022 (w), 1021 (w), 885 (w), 835 (w), 834 (w), 833 (w), 742 (m), 741 (m).

HRMS (ESI) calcd. for $C_{40}H_{50}N_3O_6Si^+$ $[M+H]^+$ 696.3463; found 696.3462.

(*S*)-Methyl 2-((*S*)-2-(((benzyloxy)carbonyl)amino)-3-hydroxypropanamido)-3-(2-((triisopropylsilyl)ethynyl)-1*H*-indol-3-yl)propanoate (5e)

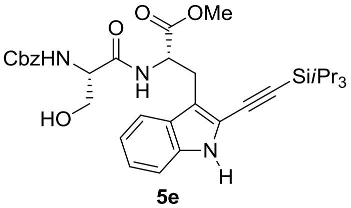

5e

Starting from peptide **4e** (88 mg, 0.20 mmol) alkyne **5e** (79.1 mg, 0.128 mmol, 64% yield) was obtained as a white solid.

Rf: 0.60 (hex:EtOAc 2:3).

Mp: 65-67 °C.

^{1}H NMR (400 MHz, $CDCl_3$) δ 8.15 (s, 1 H, indole NH), 7.50 (d, 1 H, *J* = 8.0 Hz, ArH), 7.38-7.20 (m, 7 H, ArH), 7.12 (m, 1 H, ArH), 6.73 (d, 1 H, *J* = 7.5 Hz, NH), 5.57 (d, 1 H, *J* = 7.4 Hz, NH), 5.06 (s, 2 H, Cbz CH_2), 4.88 (m, 1 H, CH), 4.17 (m, 1 H, CH), 3.91 (m, 1 H, CH_2O), 3.71 (s, 3 H, OMe), 3.51 (m, 1 H, CH_2O), 3.40 (dd, 1 H, *J* = 14.4, 5.8 Hz, CH_2), 3.31 (dd, 1 H, *J* = 14.4, 7.7 Hz, CH_2), 2.79 (t, 1 H, *J* = 6.9 Hz, OH), 1.37-0.97 (m, 21 H, TIPS).

^{13}C NMR (101 MHz, $CDCl_3$) δ 172.4, 170.5, 156.0, 136.2, 135.5, 128.6, 128.3, 128.1, 126.7, 124.2, 120.6, 118.9, 118.3, 116.5, 111.2, 99.0, 97.3, 67.1, 63.3, 55.5, 53.3, 52.9, 27.5, 18.7, 11.3.

IR 3398 (m), 3322 (m), 3321 (m), 3320 (m), 3319 (m), 3062 (w), 3061 (w), 2947 (m), 2946 (m), 2893 (w), 2892 (w), 2866 (m), 2865 (m), 2147 (w), 1724 (s), 1723 (s), 1669 (s), 1511 (s), 1505 (s), 1504 (s), 1460 (m), 1455 (m), 1350 (m), 1221 (s), 1118 (w), 1063 (m), 1021 (m), 884 (m), 742 (s), 727 (s), 726 (s).

HRMS (ESI) calcd. for $C_{34}H_{46}N_3O_6Si^+$ $[M+H]^+$ 620.3150; found 620.3146.

(*S*)-Benzyl 2-(((*S*)-1-methoxy-1-oxo-3-(2-((triisopropylsilyl)ethynyl)-1*H*-indol-3-yl)propan-2-yl)carbamoyl)pyrrolidine-1-carboxylate (5f)

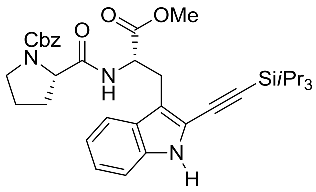

5f

Starting from peptide **4f** (90 mg, 0.20 mmol) alkyne **5f** (66.5 mg, 0.106 mmol, 53% yield) was obtained as a white solid.

Rf=0.30 (hex/EtOAc 3/2).

Mp: 58-68 °C.

^{1}H NMR (400 MHz, $CDCl_3$) δ 8.09 (s, 1 H, indole NH), 7.55-7.49 (m, 1 H, ArH), 7.37-6.98 (m, 8 H, ArH), 6.40 (br s, 0.5 H, NH rotamer 1), 5.22-5.02 (m, 1.5 H, NH), 5.10 (s, 1 H, Cbz CH_2 rotamer 1), 5.10 (s, 1 H, Cbz CH_2 rotamer 2), 4.99-4.77 (m, 1 H, CH), 4.29 (br s, 0.5 H, CH, rotamer 1), 4.22 (br s, 0.5 H, CH, rotamer 2), 3.64 (s, 1.5 H, OMe, rotamer 1), 3.62 (s, 1.5 H, OMe, rotamer 2), 3.46-3.12 (m, 4 H, CH_2), 2.32-1.38 (m, 4 H, CH_2), 1.40-0.88 (m, 21 H, TIPS). Broad peaks were observed due to the presence of rotamers. Rotamers are not specifically assigned.

^{13}C NMR (101 MHz, $CDCl_3$) δ 172.1, 172.0, 171.4, 155.9, 155.0, 136.6, 135.5, 128.5, 128.0, 127.9, 127.1, 124.1, 123.9, 121.5, 120.7, 120.5, 120.4, 119.3, 119.3, 119.1, 119.0, 118.1, 117.4, 117.1, 110.9, 110.9, 98.5, 97.5, 97.4, 67.2, 60.8, 60.5, 53.1, 52.9, 52.4, 47.4, 46.9, 30.8, 28.4, 28.0, 24.3, 23.4, 18.7, 11.3. Due to the presence of rotamers, not all peaks can be resolved.

IR 3327 (w), 3307 (w), 3302 (w), 3301 (w), 3300 (w), 2946 (w), 2945 (w), 2889 (w), 2865 (w), 2250 (w), 2146 (w), 1743 (m), 1688 (m), 1687 (m), 1517 (w), 1437 (m), 1436 (m), 1414 (m), 1353 (m), 1280 (w), 1242 (w), 1241 (w), 1212 (m), 1211 (m), 1181 (m), 1180 (m), 1119 (m), 1090 (w), 994 (w), 913 (s), 883 (m), 738 (s).

HRMS (ESI) calcd for $C_{36}H_{48}N_3O_5Si^+$ $[M+H]^+$ 630.3358; found 630.3343.

(5*S*,8*S*,11*S*)-Methyl 5,11-diisopropyl-3,6,9-trioxo-1-phenyl-8-((2-((triisopropylsilyl)ethynyl)-1*H*-indol-3-yl)methyl)-2-oxa-4,7,10-triazadodecan-12-oate (5h)

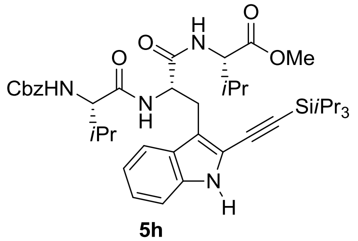

5h

Starting from peptide **4h** (86 mg, 0.16 mmol) alkyne **5h** (57 mg, 0.078 mmol, 50% yield) was obtained as a white solid.

Rf= 0.75 (hex/EtOAc 2:3).

Mp 77-79 °C.

^{1}H NMR (400 MHz, $CDCl_3$) δ 8.09 (s, 1 H, indole NH), 7.63 (d, 1 H, *J* = 8.0 Hz, ArH), 7.42-7.29 (m, 5 H, ArH), 7.26-7.17 (m, 2 H, ArH), 7.11 (m, 1 H, ArH), 6.50 (d, 1 H, *J* = 7.1 Hz, NH), 6.23 (d, 1 H, *J* = 8.4 Hz, NH), 5.16 (d, 1 H, *J* = 8.1 Hz, NH), 5.08 (m, 2 H, Cbz CH_2), 4.69 (dd, 1 H, *J* = 7.6, 7.6 Hz, CH), 4.41 (dd, 1 H, *J* = 8.3, 5.1 Hz, CH), 4.01 (m, 1 H, CH), 3.60 (s, 3 H, OMe), 3.30 (d, 2 H, *J* = 7.7 Hz, CH_2), 2.04 (m, 2 H, Val CH), 1.21-1.03 (m, 21 H, TIPS), 0.86 (d, 3 H, *J* = 6.8 Hz, Val CH_3), 0.81 (d, 3 H, *J* = 7.8 Hz, Val CH_3), 0.80 (d, 3 H, *J* = 7.4 Hz, Val CH_3), 0.68 (d, 3 H, *J* = 6.6 Hz, Val CH_3).

^{13}C NMR (101 MHz, $CDCl_3$) δ 171.4, 171.1, 170.5, 156.2, 136.3, 135.7, 128.6, 128.3, 128.1, 126.8, 124.2, 120.6, 119.2, 118.2, 117.3, 111.0, 98.8, 97.3, 67.1, 60.2, 57.4, 54.0, 52.0, 31.6, 31.2, 27.9, 19.1, 18.7, 17.9, 17.1, 11.3. One valine CH_3 is not resolved.

IR 3313 (m), 3063 (w), 2957 (m), 2868 (w), 2146 (w), 1708 (m), 1656 (s), 1515 (s), 1462 (m), 1346 (w), 1233 (m), 1146 (w), 1027 (w), 884 (w), 742 (s).

HRMS (ESI) calcd. for $C_{41}H_{59}N_4O_6Si^+$ $[M+H]^+$ 731.4198; found 731.4190.

5. NMR Spectra of new compounds

^{1}H NMR, 400 MHz, $CDCl_3$

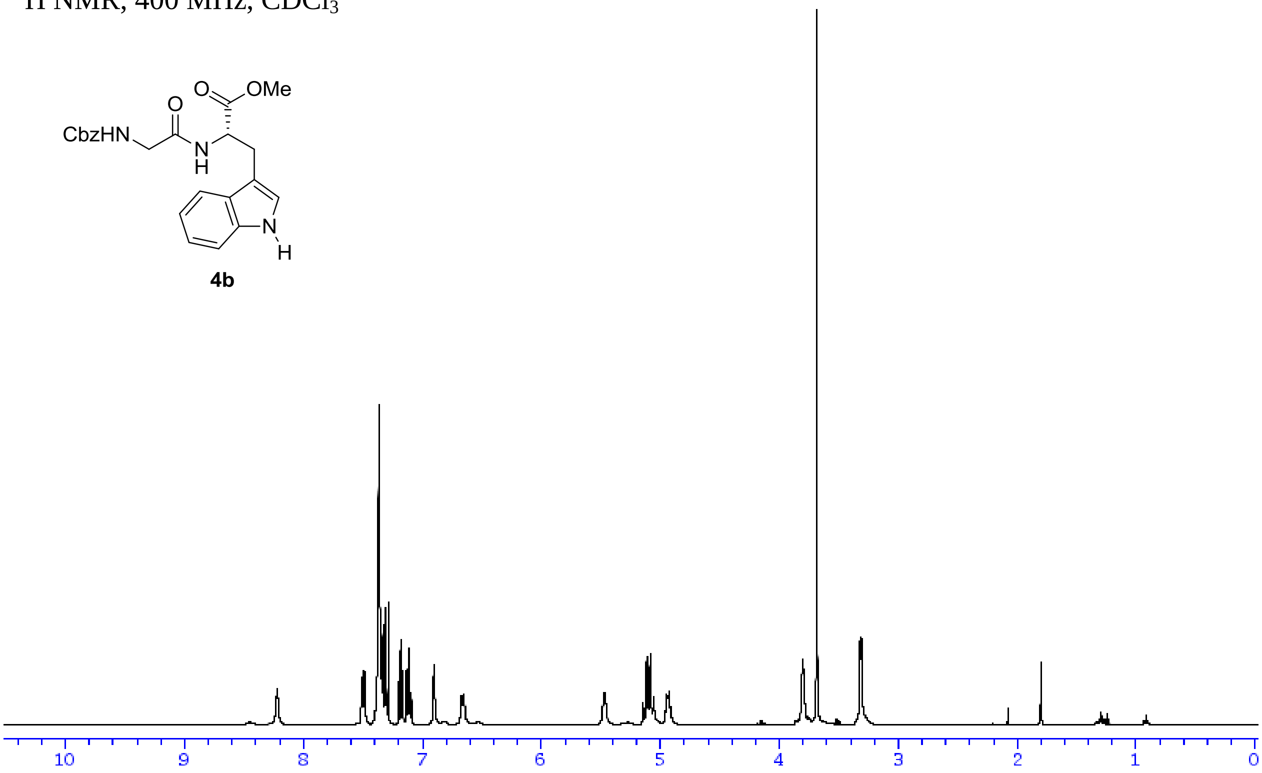

4b

^{13}C NMR, 100 MHz, $CDCl_3$

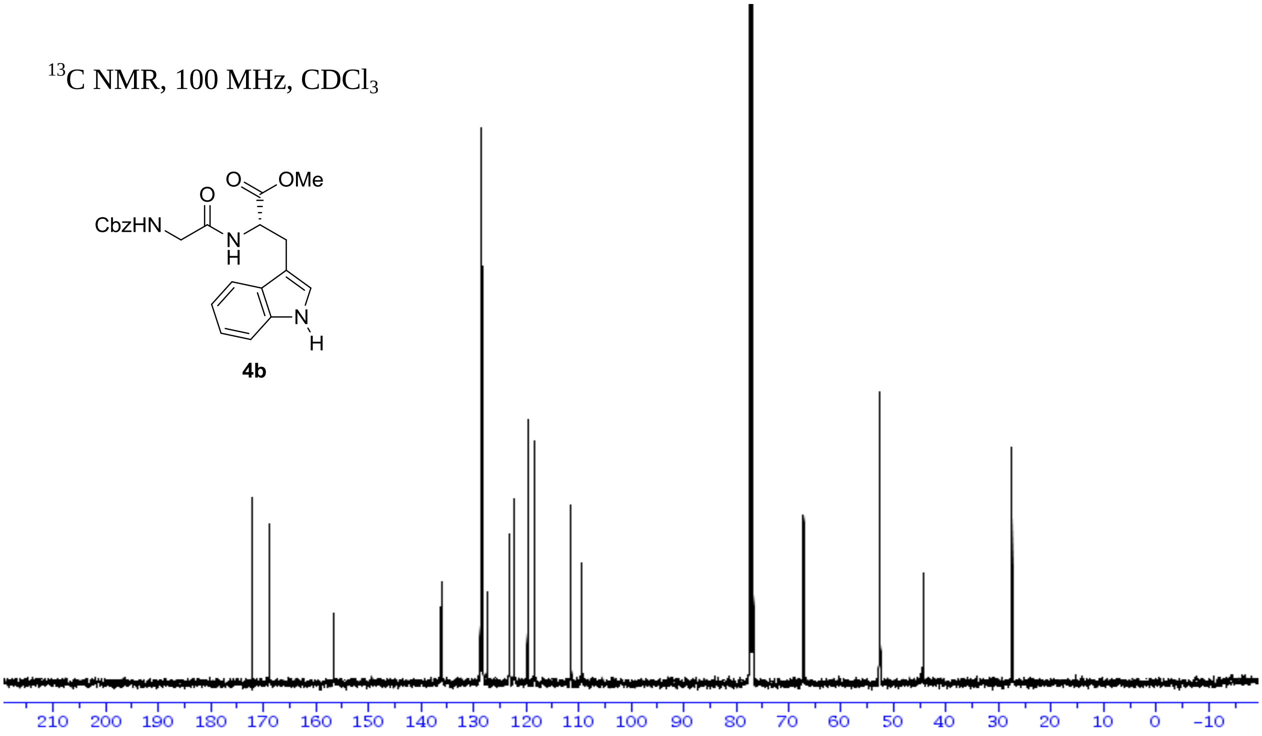

4b

^{1}H NMR, 400 MHz, $CDCl_3$

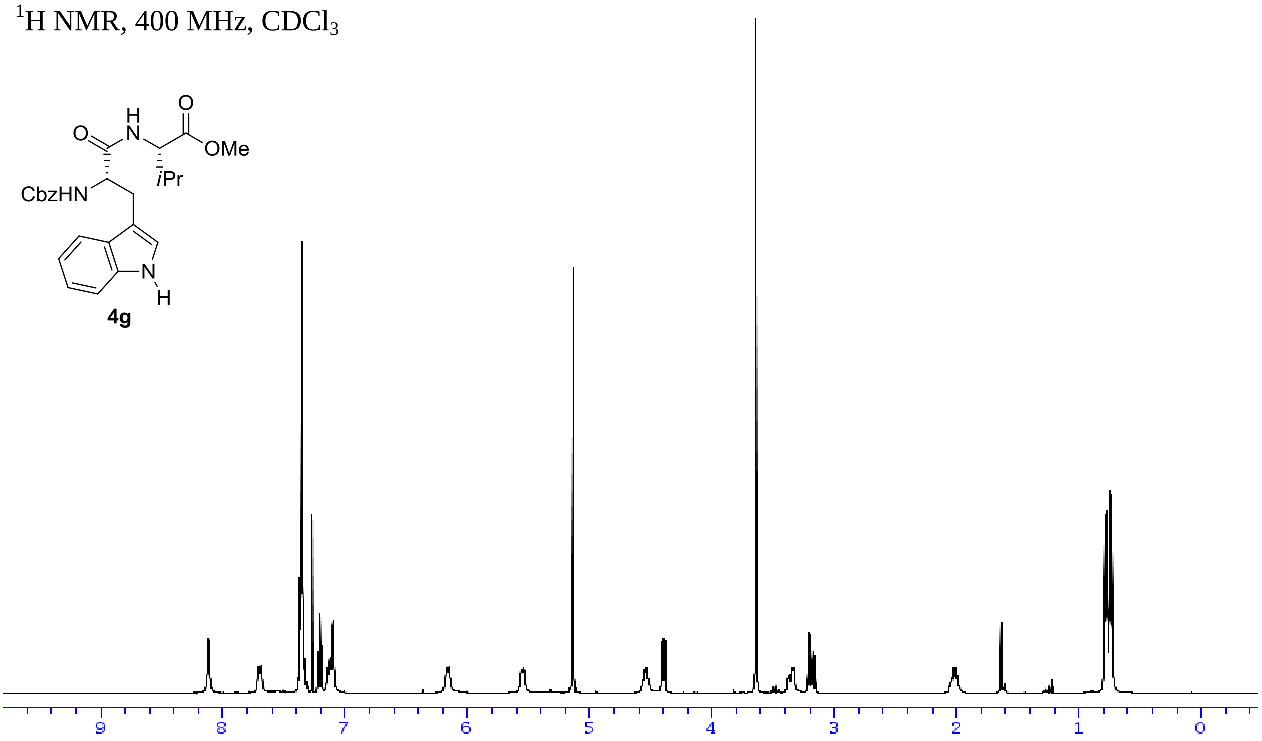

^{13}C NMR, 100 MHz, $CDCl_3$

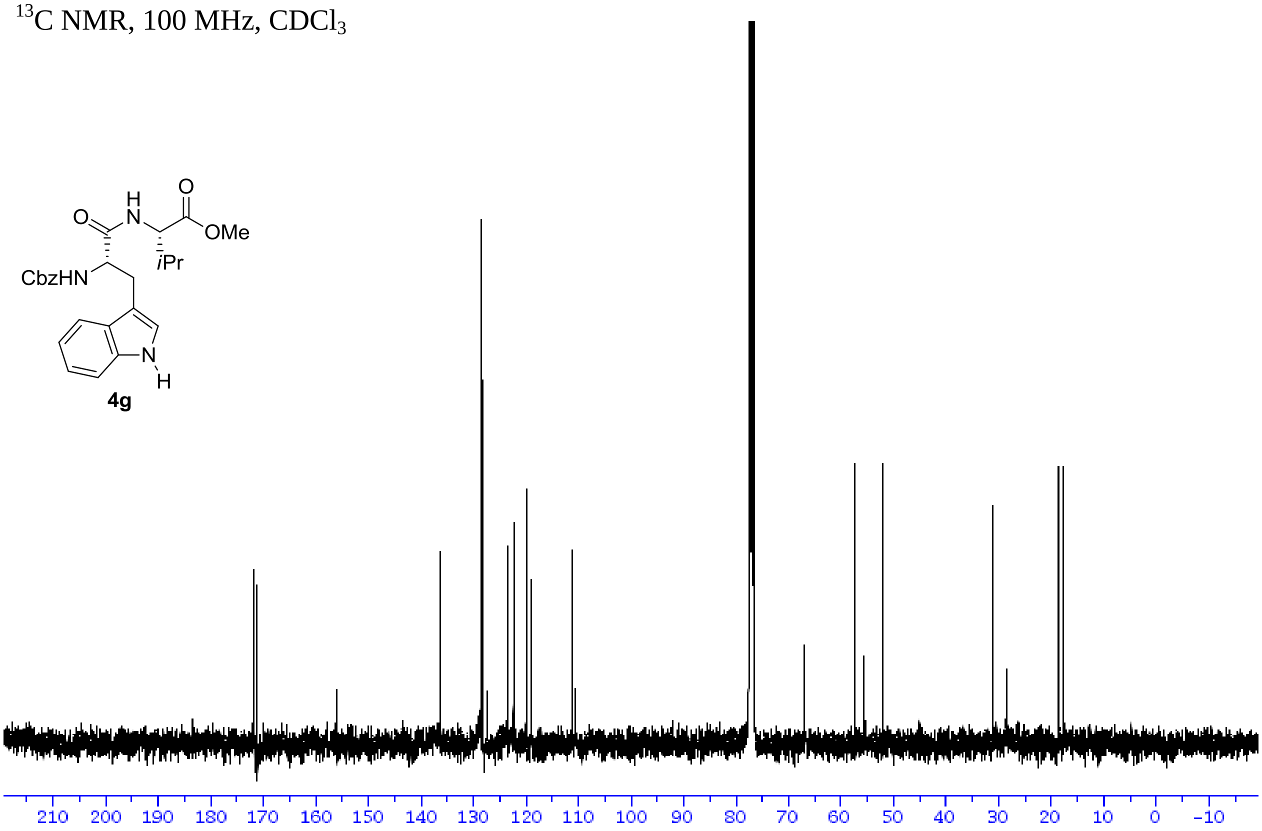

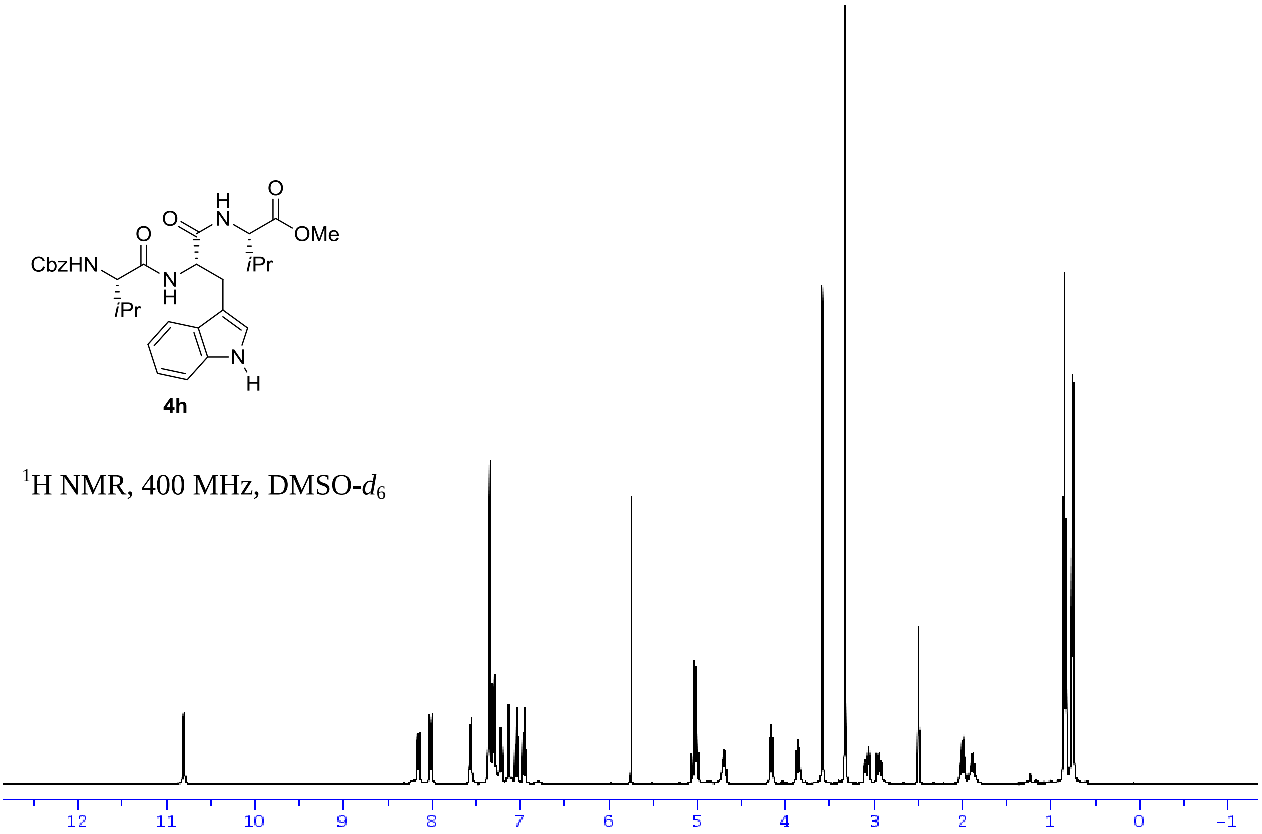

4h

^{1}H NMR, 400 MHz, DMSO-d_6

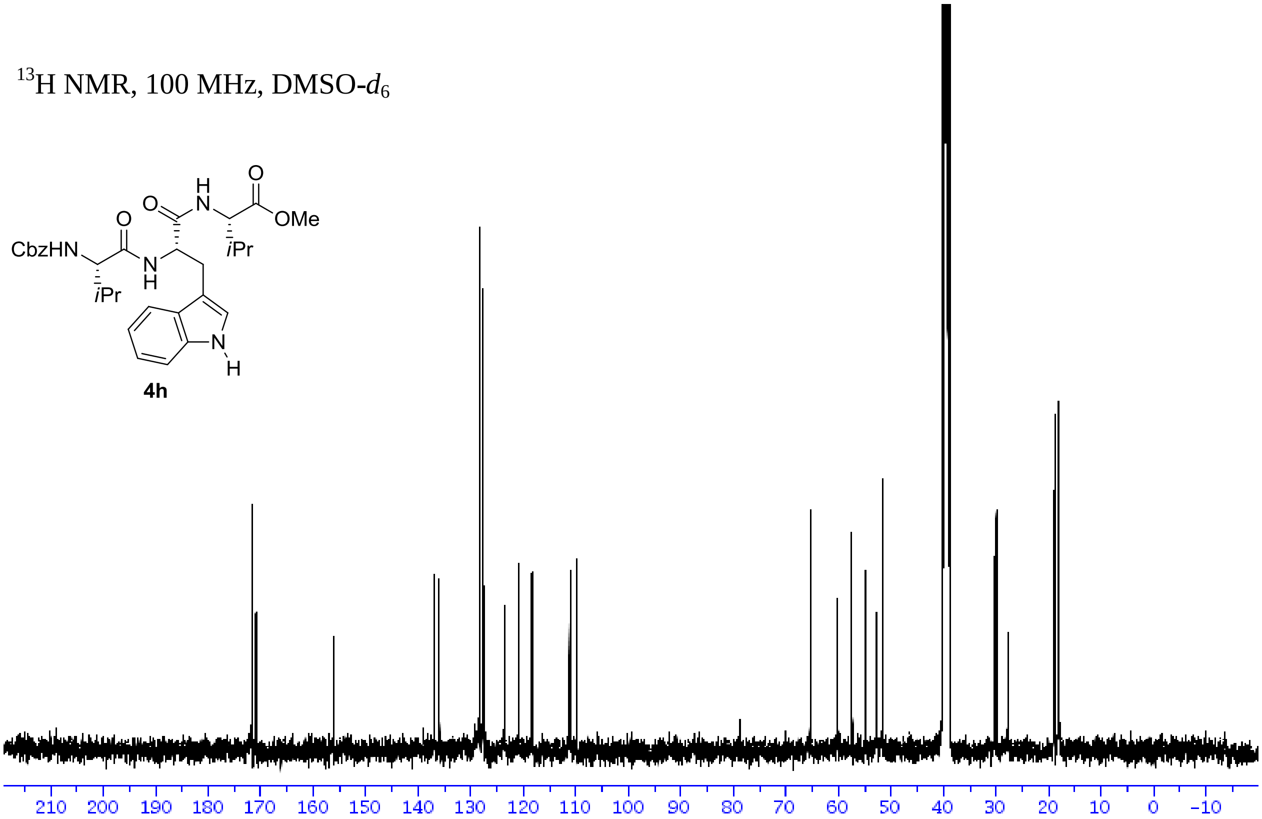

^{13}H NMR, 100 MHz, DMSO-d_6

4h

^{1}H NMR, 400 MHz, $CDCl_3$

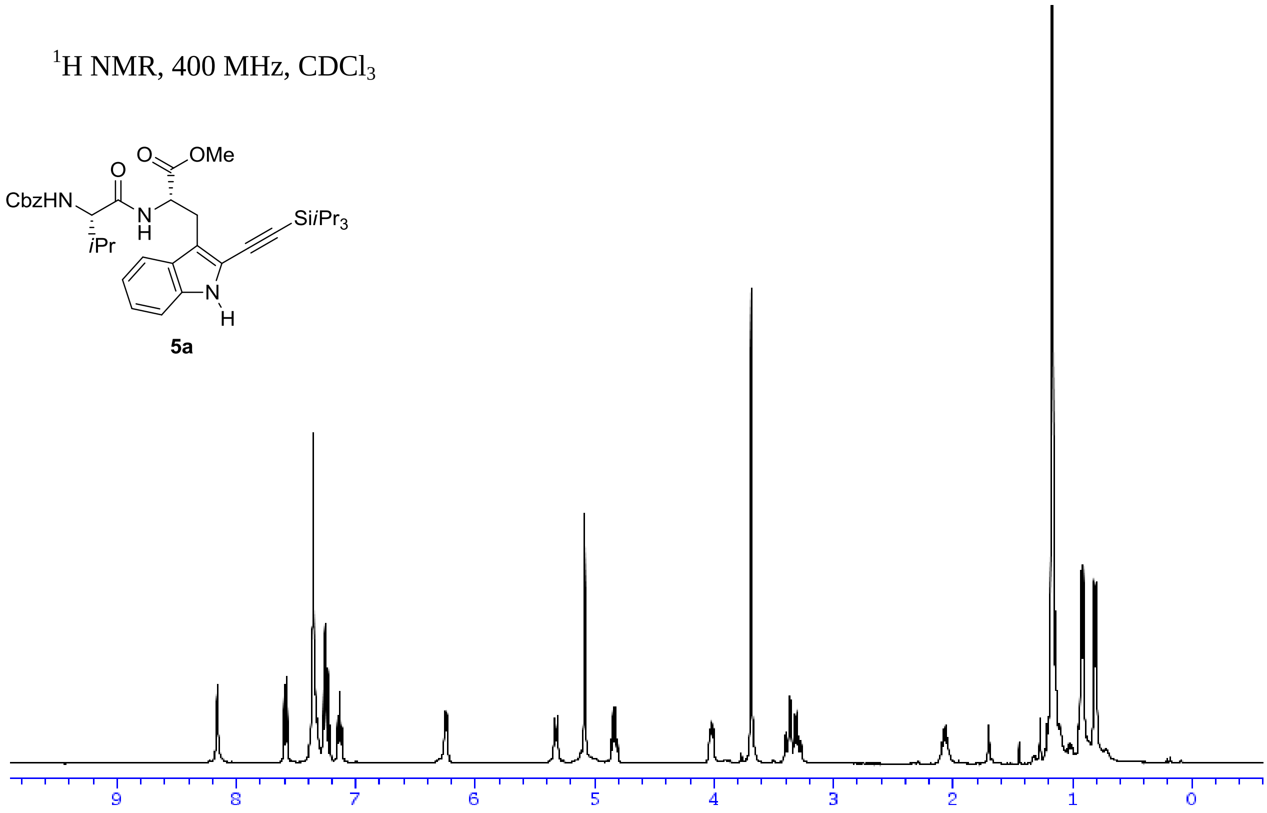

^{13}C NMR, 100 MHz, $CDCl_3$

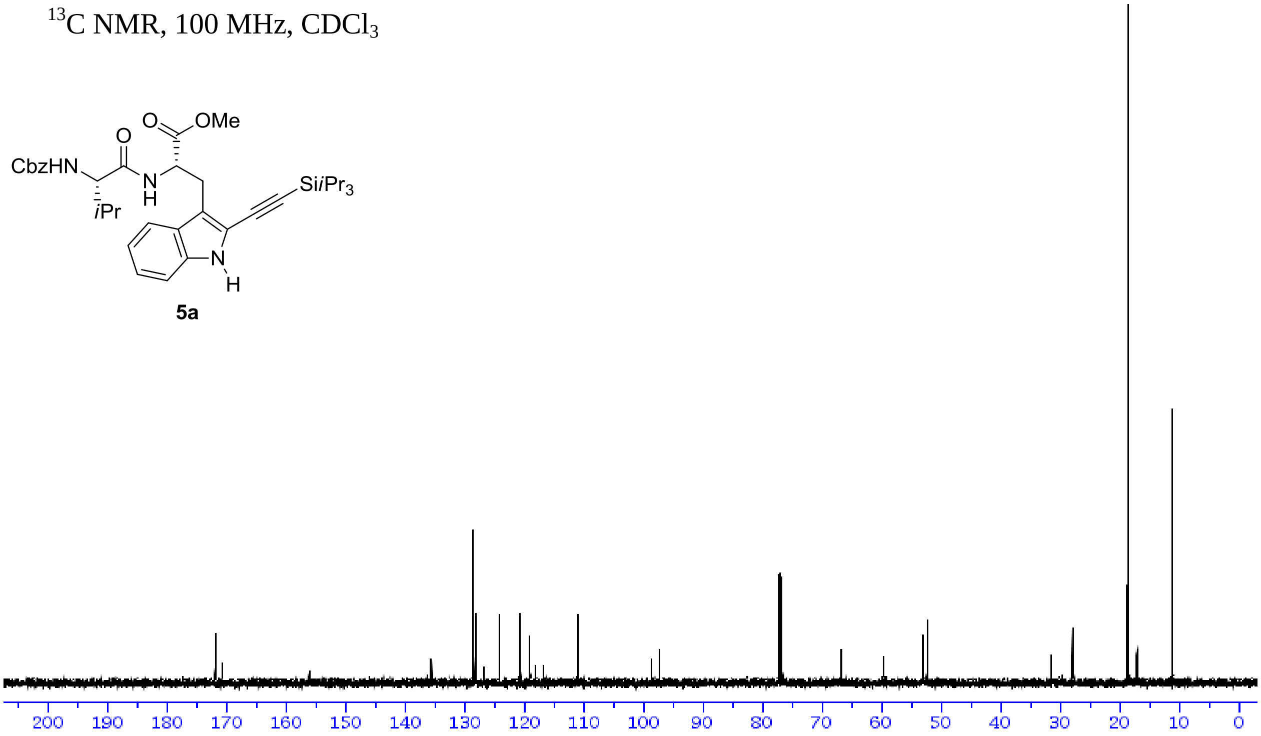

^{1}H NMR, 400 MHz, $CDCl_3$

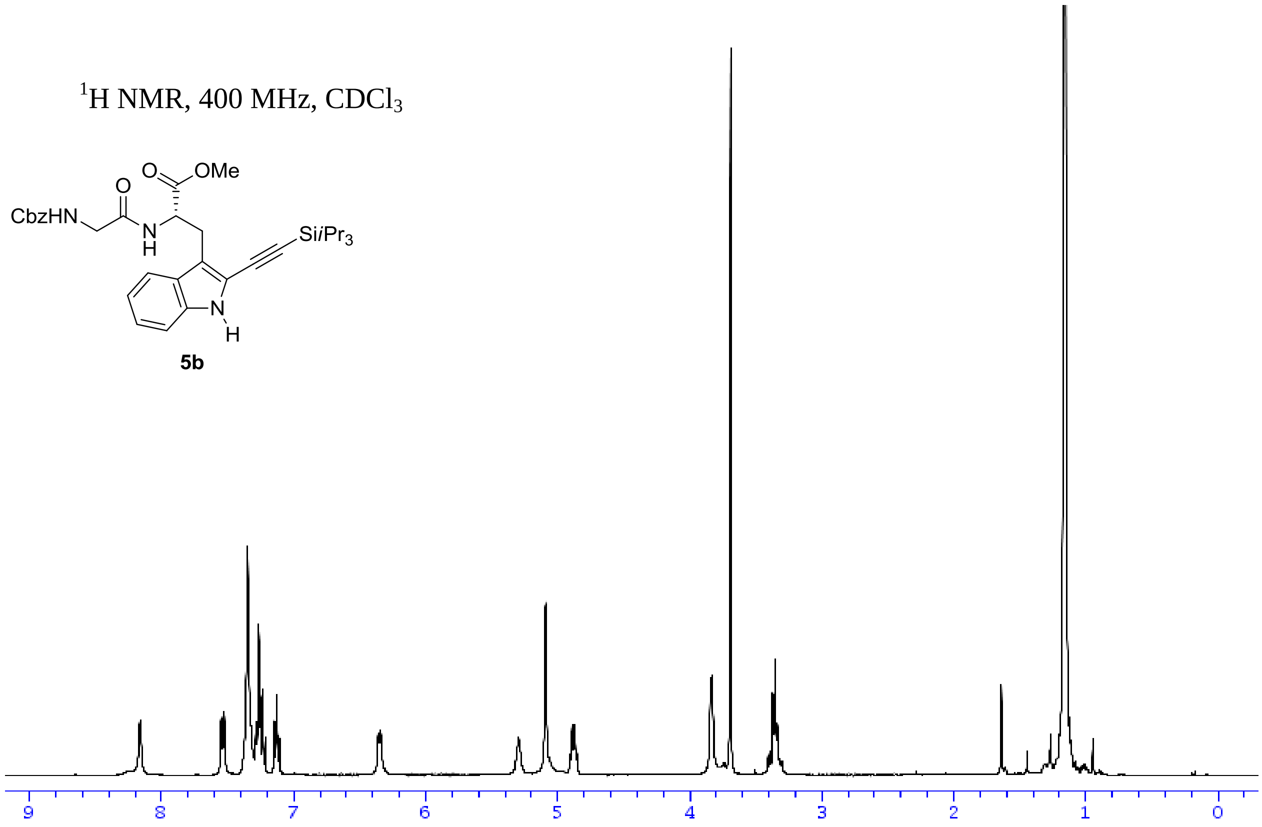

^{13}C NMR, 100 MHz, $CDCl_3$

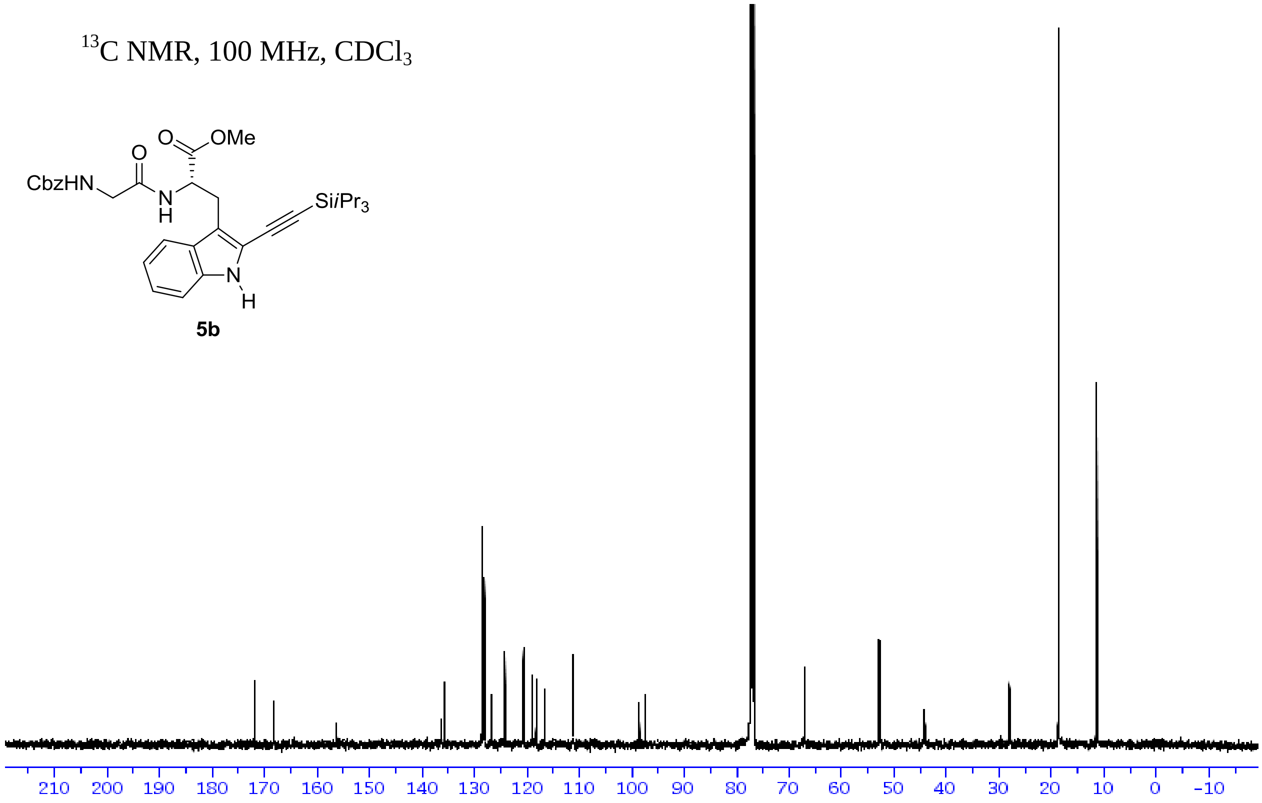

^{1}H NMR, 400 MHz, $CDCl_3$

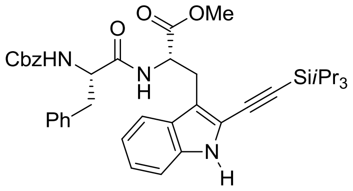

5c

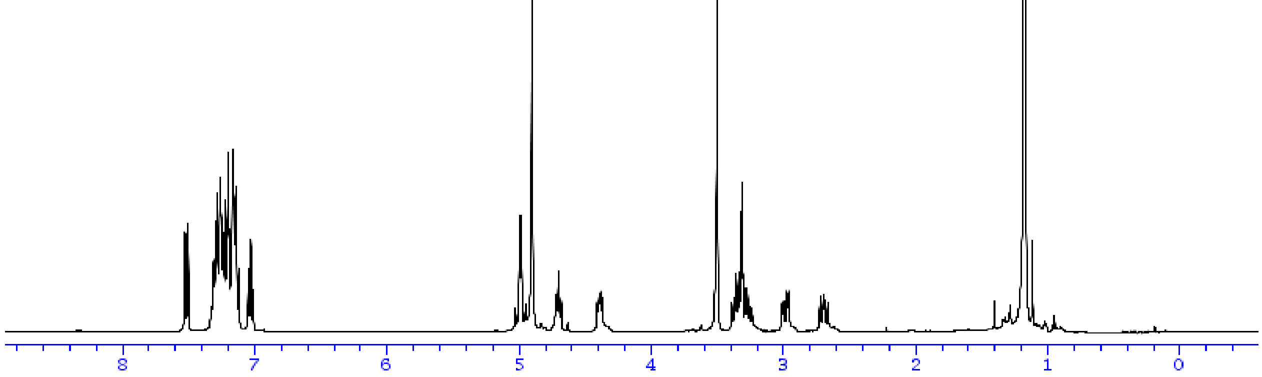

^{13}C NMR, 100 MHz, $CDCl_3$

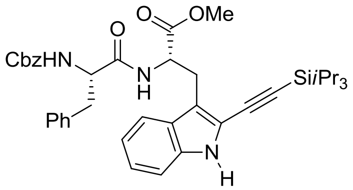

5c

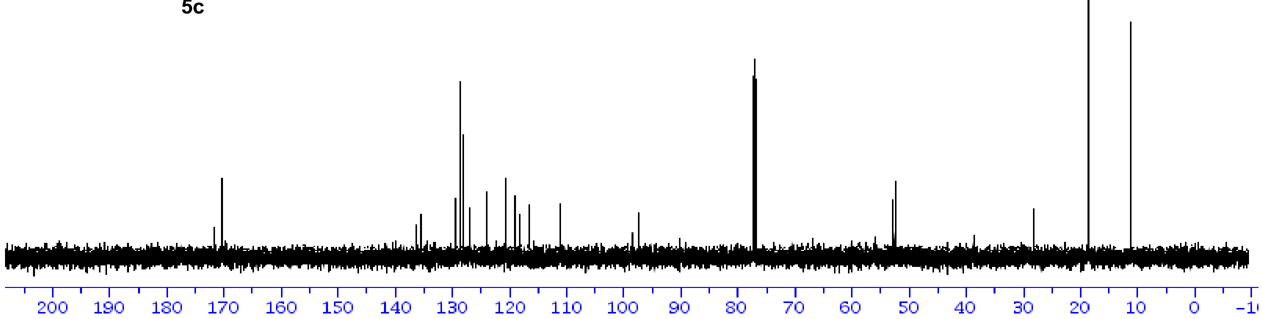

^{1}H NMR, 400 MHz, $CDCl_3$

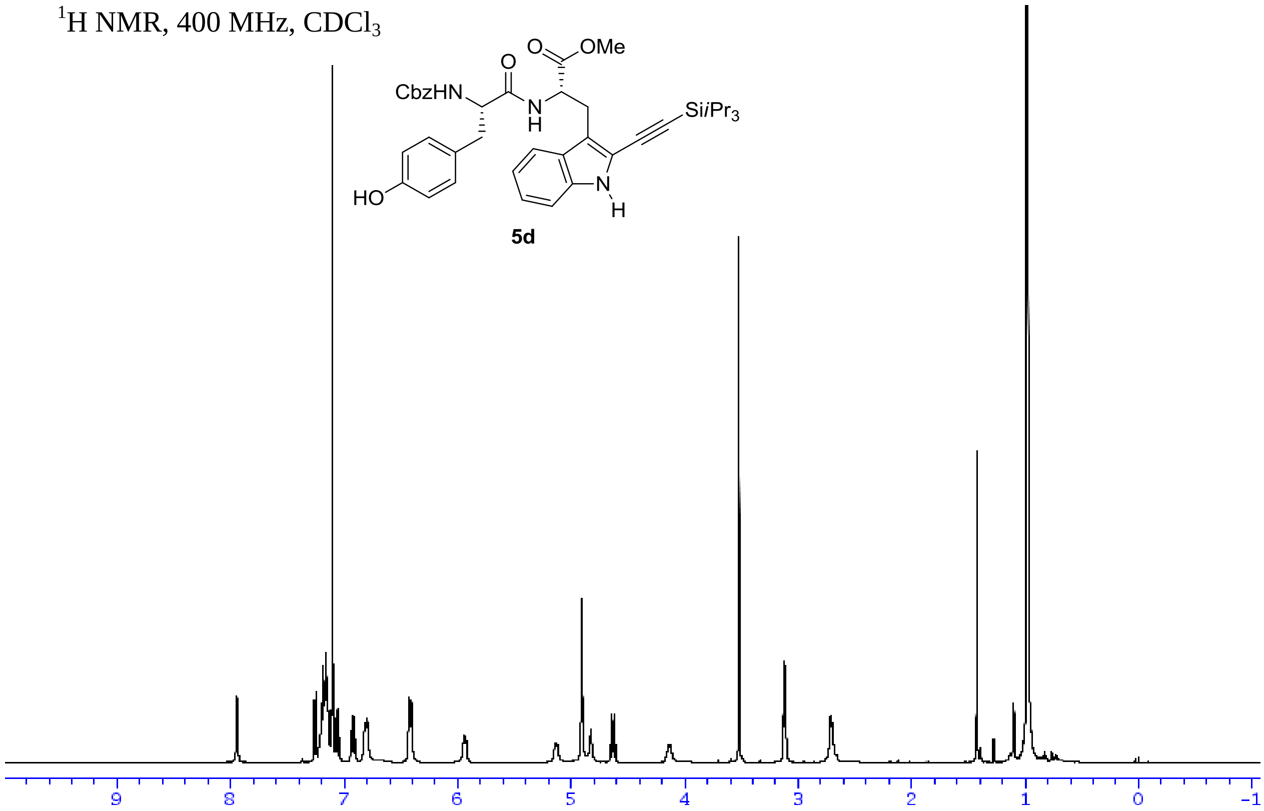

^{13}C NMR, 100 MHz, $CDCl_3$

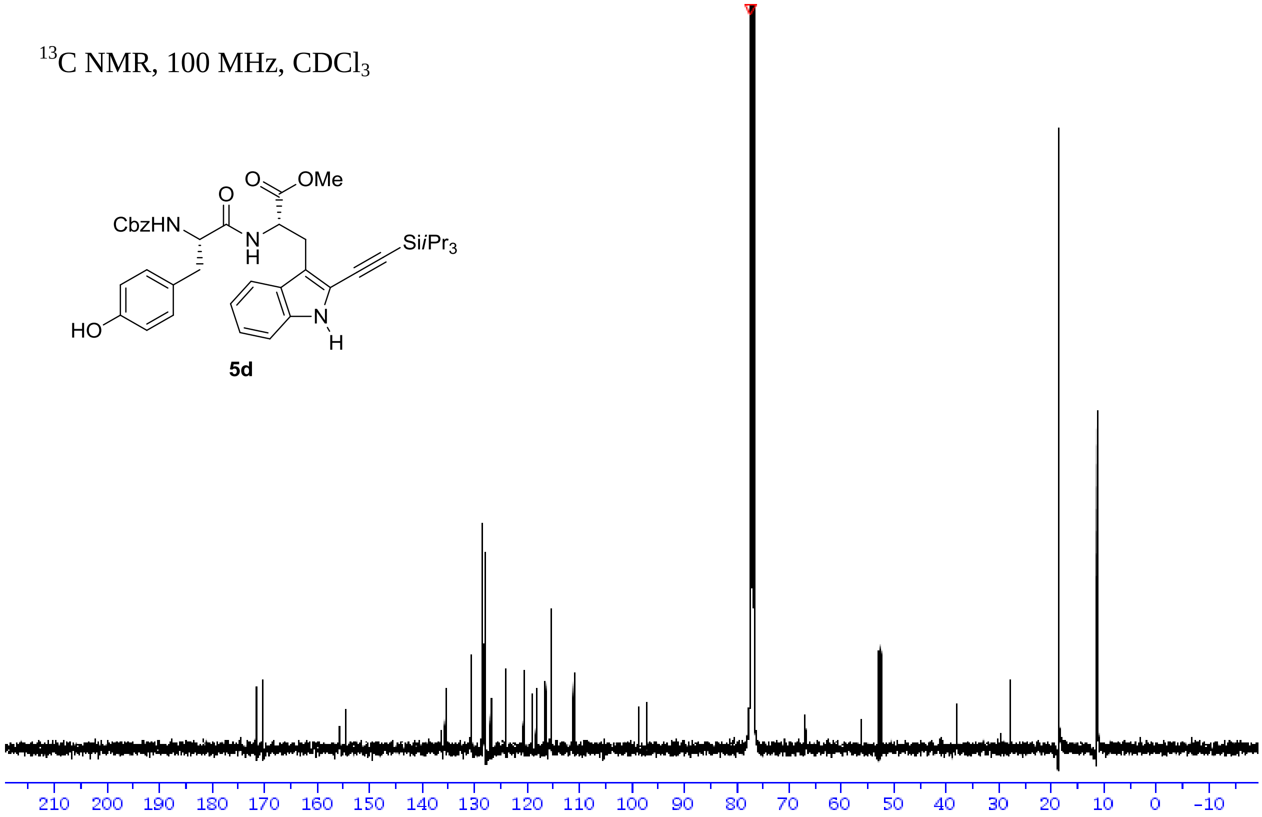

^{1}H NMR, 400 MHz, $CDCl_3$

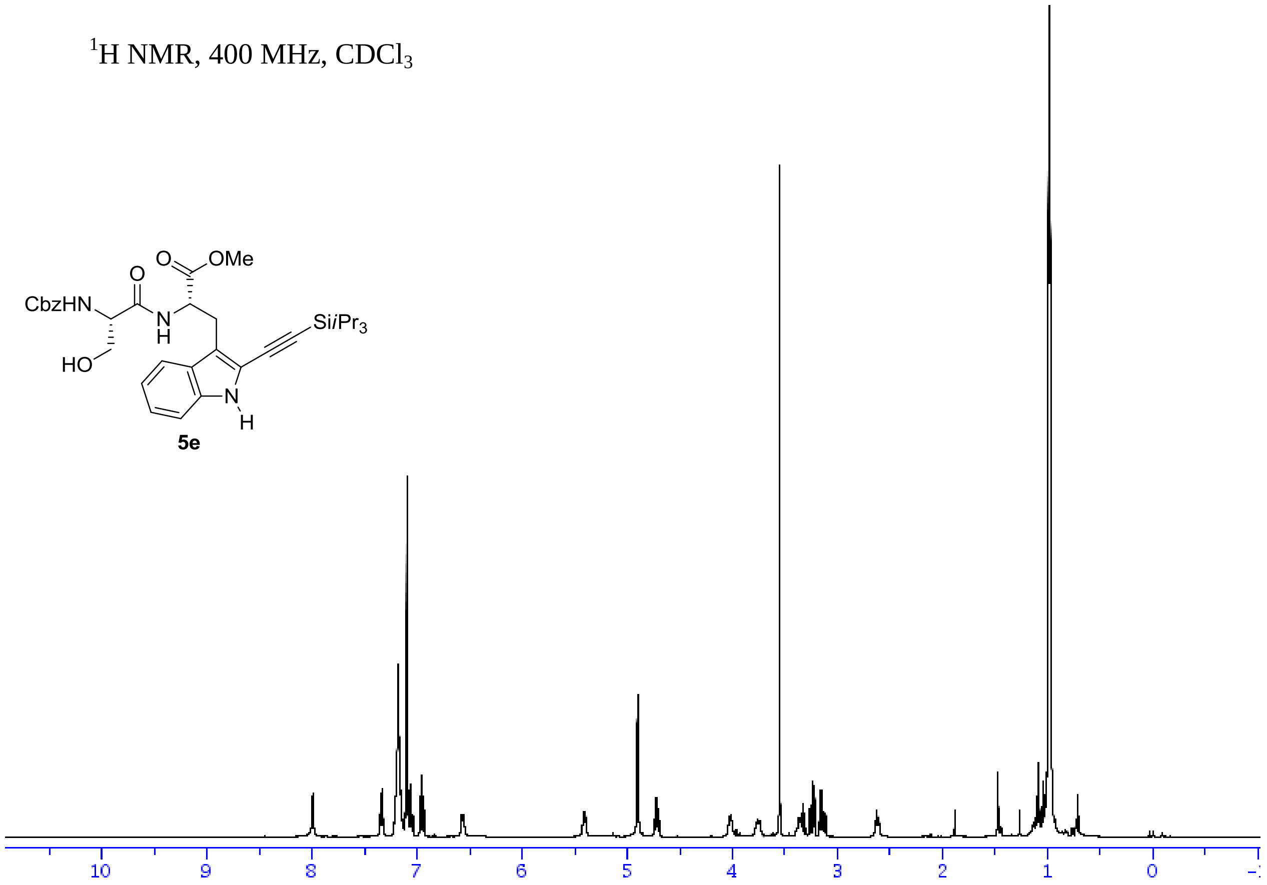

^{13}C NMR, 100 MHz, $CDCl_3$

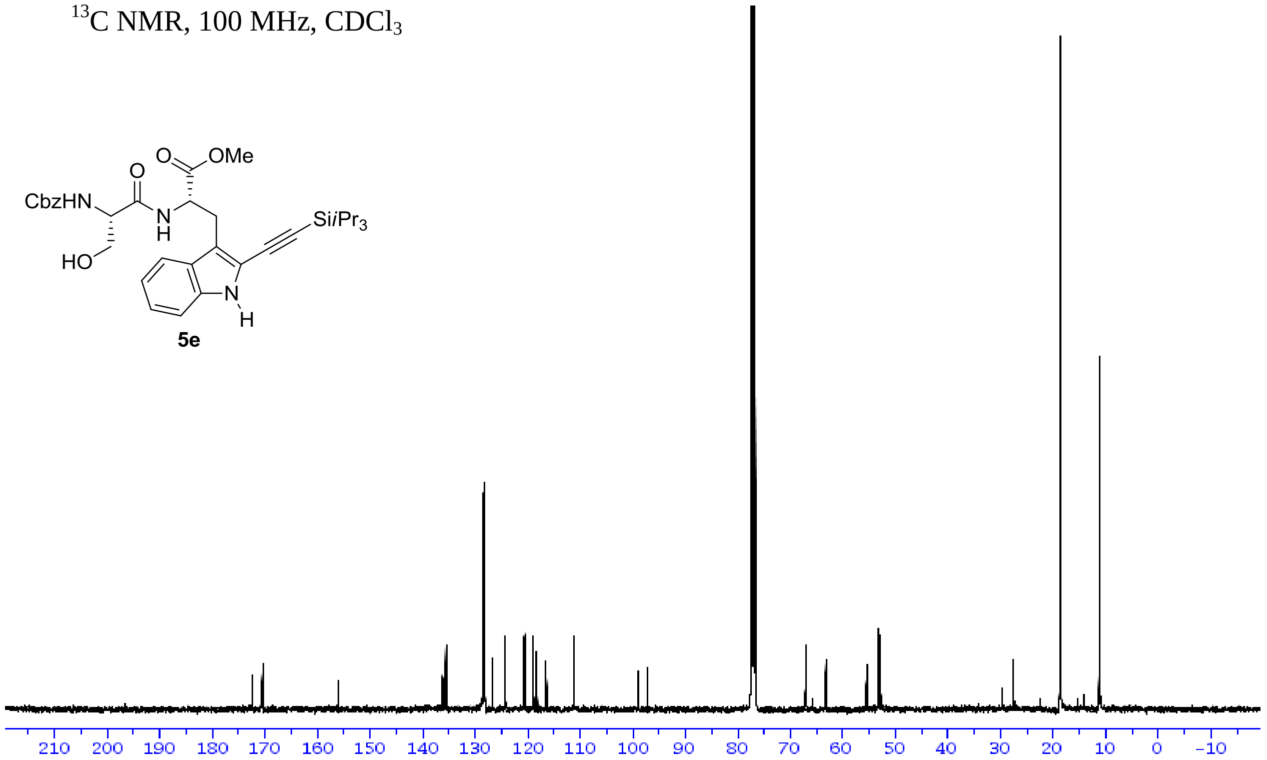

^{1}HNMR, 400 MHz, $CDCl_3$

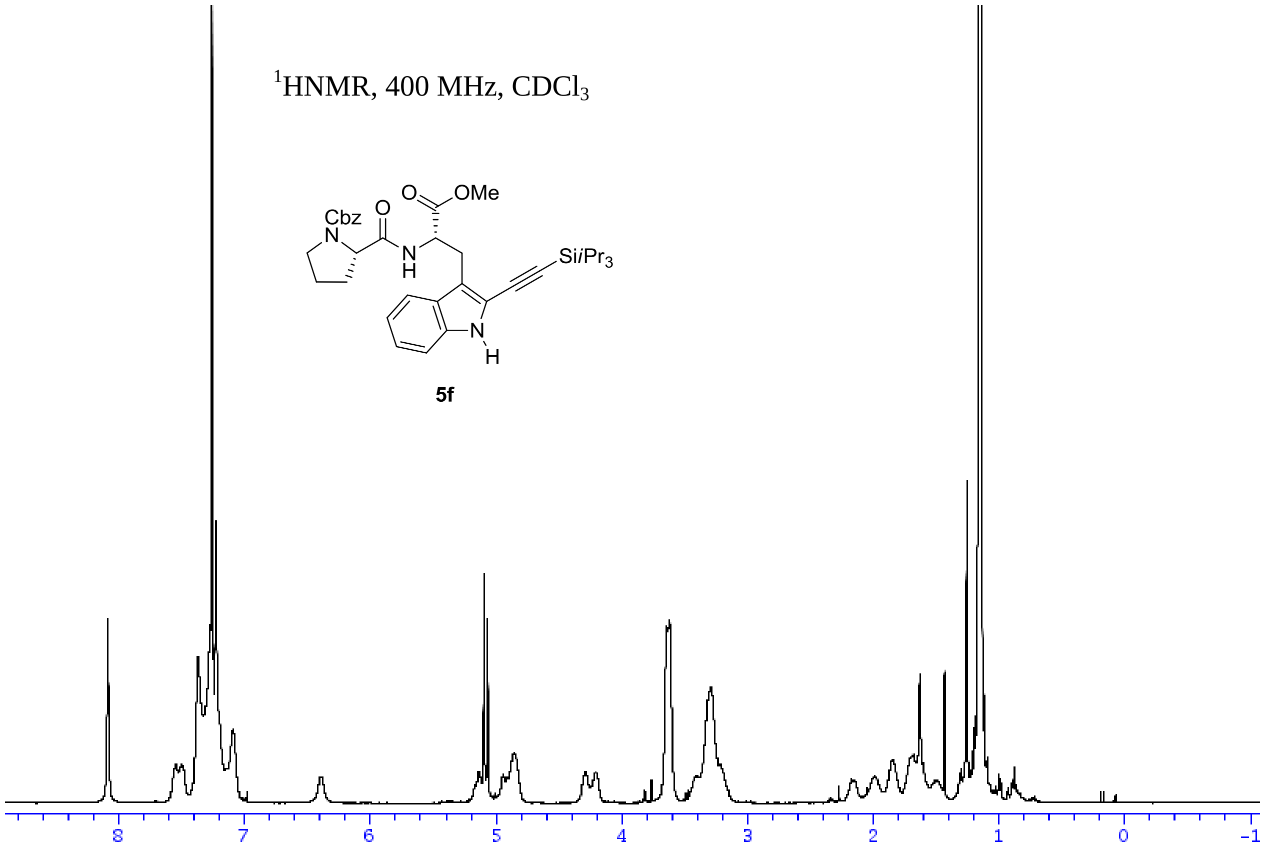

5f

^{13}C NMR, 100 MHz, $CDCl_3$

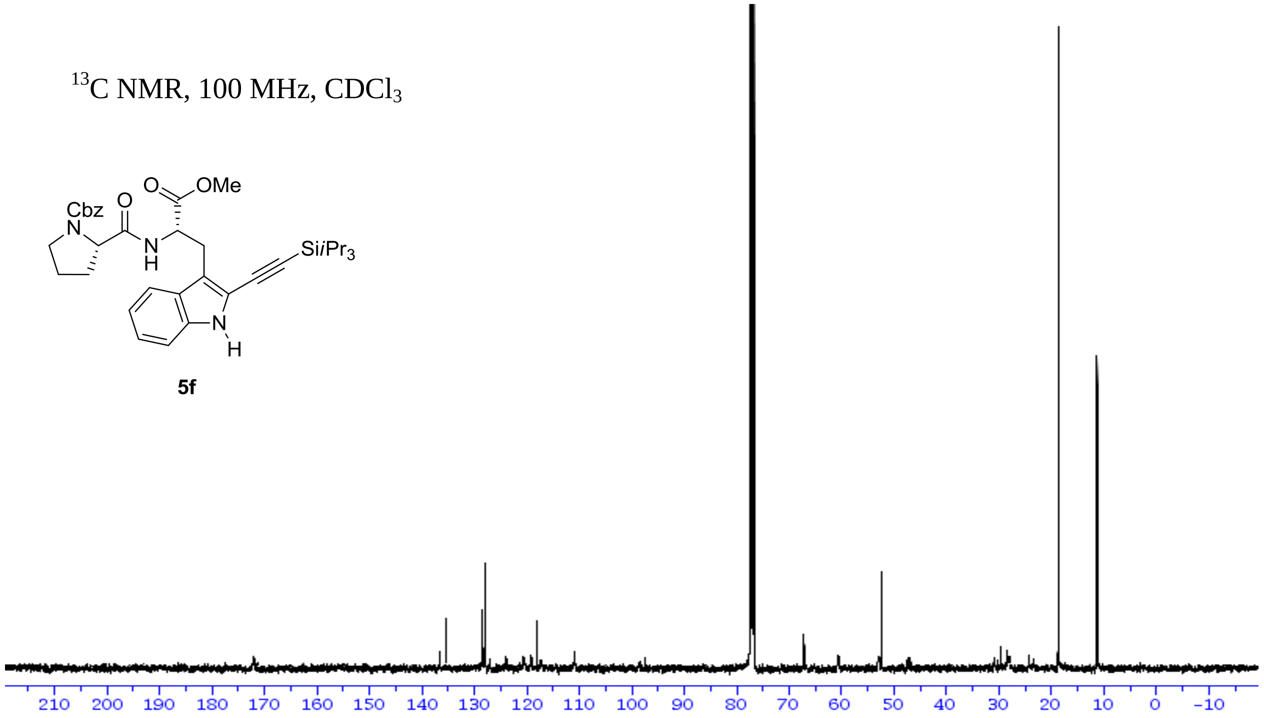

5f

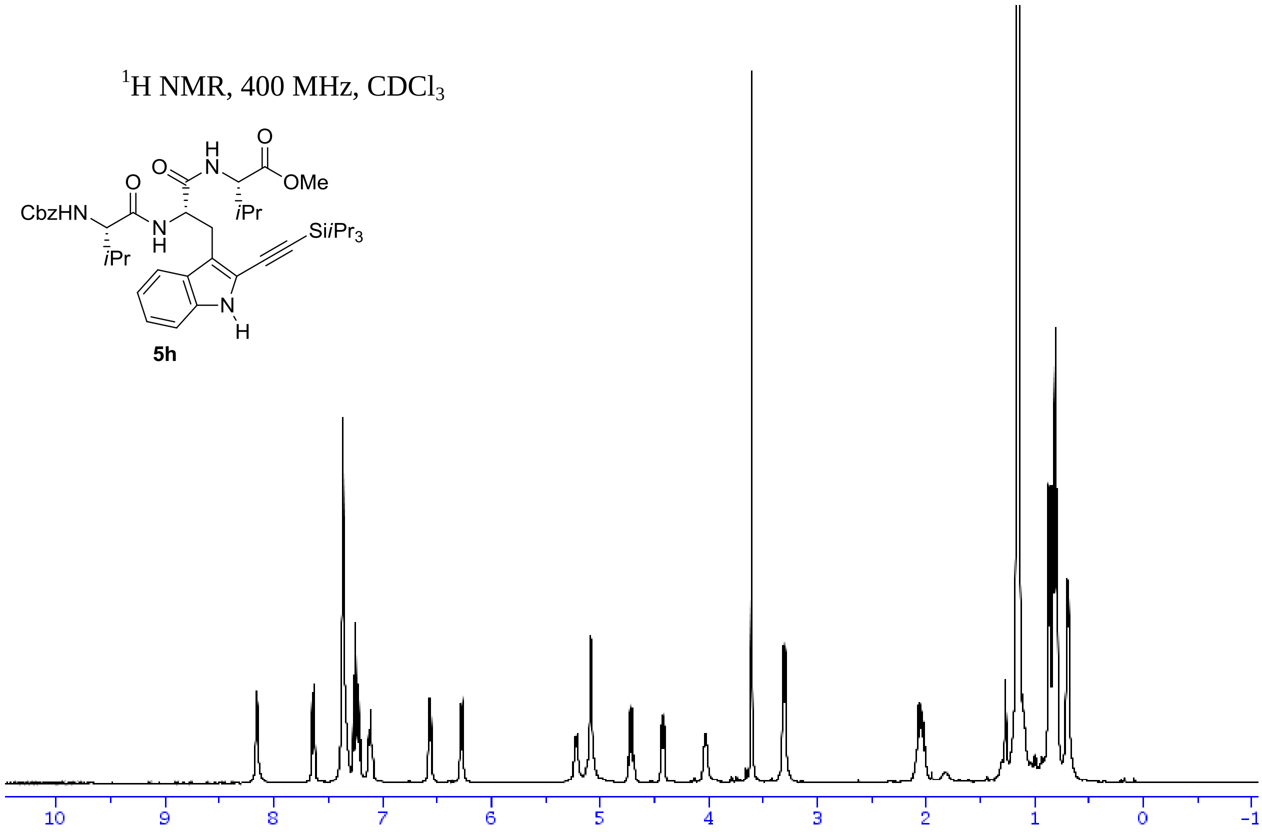
^{1}H NMR, 400 MHz, $CDCl_3$
CbzHN
O
N
H
O
H
N
O
OMe
iPr
iPr
SiiPr$_3$
N
H
5h
10 9 8 7 6 5 4 3 2 1 0 -1

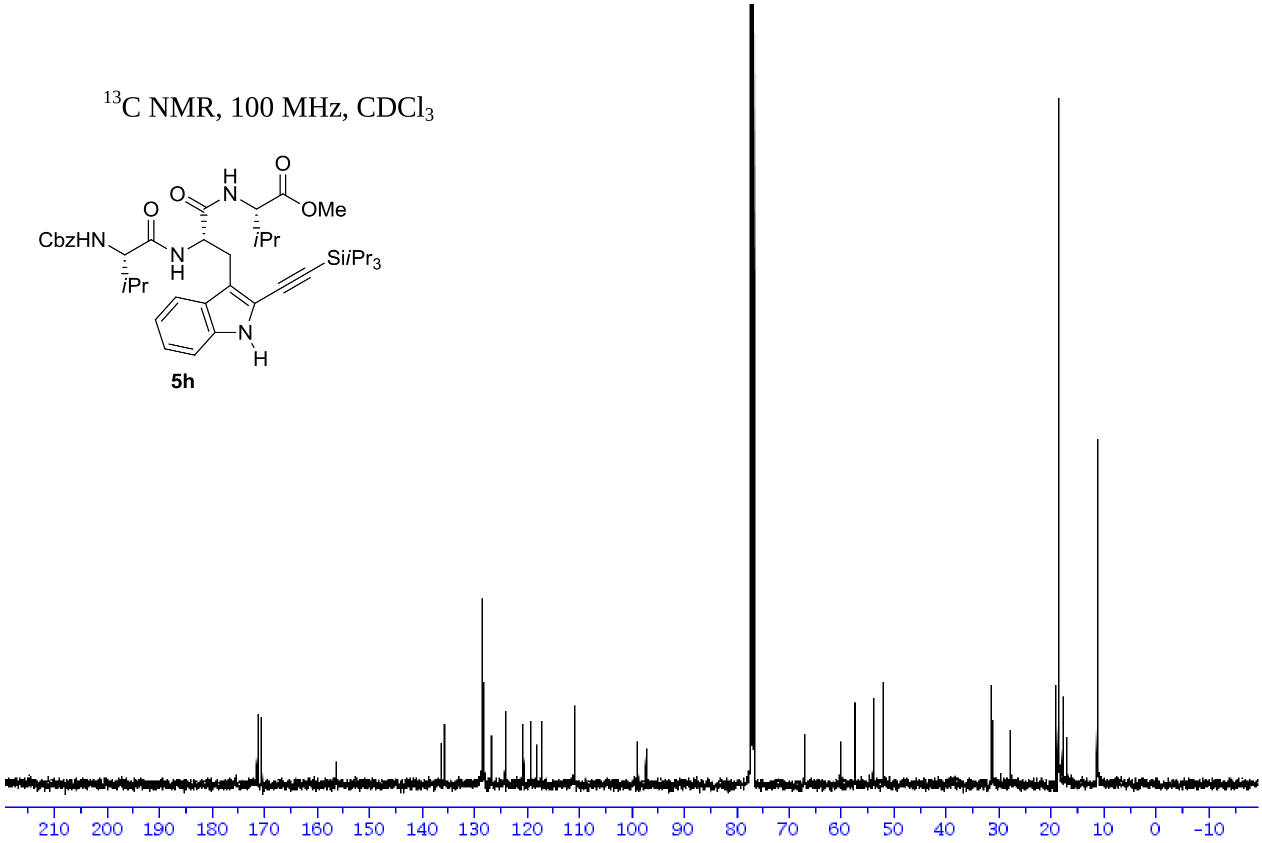
^{13}C NMR, 100 MHz, $CDCl_3$
CbzHN
O
N
H
O
H
N
O
OMe
iPr
iPr
SiiPr$_3$
N
H
5h
210 200 190 180 170 160 150 140 130 120 110 100 90 80 70 60 50 40 30 20 10 0 -10
